# The Interaction of Structural Analogues of Phenothiazines in p53-Dependent Cellular Signaling Pathways

**DOI:** 10.1021/acsomega.5c04145

**Published:** 2026-01-02

**Authors:** Klaudia Giercuszkiewicz-Haśnik, Paulina Pawicka, Małgorzata Jeleń, Beata Morak-Młodawska, Magdalena Skonieczna

**Affiliations:** † Department of Systems Biology and Engineering, 49569 Silesian University of Technology, Akademicka Street 16, Gliwice 44−100, Poland; ‡ Centre of Biotechnology, Silesian University of Technology, Krzywoustego Street 8, Gliwice 44-100, Poland; § Faculty of Medical Sciences in Katowice, Medical University of Silesia, Medyków Street 18, Katowice 40-752, Poland; ∥ Department of Organic Chemistry, Faculty of Pharmaceutical Sciences in Sosnowiec, Medical University of Silesia in Katowice, Jagiellońska Street 4, Sosnowiec 41-200, Poland

## Abstract

The cytostatic effect of structural analogues of phenothiazines, in which benzene rings were replaced by azine systems (pyridines – **BM1**, **BM2**, and quinoline – **MJ1**, **MJ2**), on the HCT116 colon cancer cell line with wild-type p53 protein status and with mutated p53 protein, was studied. The participation of the tested substances in p53-dependent cell signaling pathways was assessed. Based on the performed colorimetric MTT assays, IC_50_ values for the tested chemical compounds were calculated. The best IC_50_ values for all lines were found for the **MJ2** compound. For the HCT116 line with wild-type p53 protein, IC_50_ = 36.37 μM, and IC_50_ = 57.82 μM for HCT116 with mutated p53 protein were estimated. 72 h microscopic observations of the survival of HCT116 line cells after exposure to the tested analogues were performed, which revealed apoptotic changes in the cells. Real-time polymerase chain reaction was carried out for marker genes from p53-dependent pathways. The studies were conducted under the reference gene RPL41 for the tested genes, which included apoptosis-inducing factor mitochondria-associated 2 (AIFM2), blocker of programmed apoptotic cell death (BCL2), and primary negative regulatory factor of the p53 protein (MDM2), respectively. Two HCT116 lines were used to compare the effect related to cellular p53 protein status on the relative increase in tested gene expression. The modulation of p53 protein-dependent signaling pathways by the tested phenothiazine analogues was confirmed.

## Graphical abstract

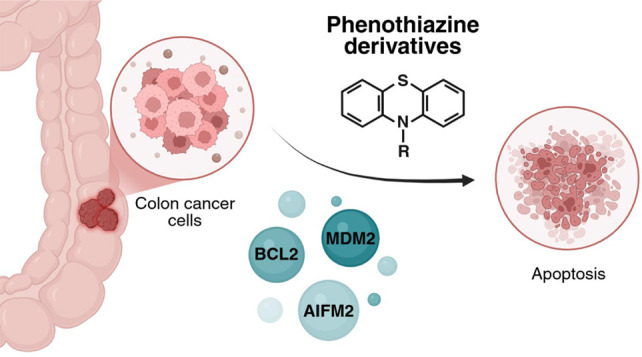

## 1 Introduction

The functioning of the human body is a very dynamic process, in which cells constantly die and new ones are created. Unfortunately, during this phenomenon, cells can also undergo mutations, which is the basis of cancer development. More than half of human cancers contain many different mutations of the TP53 gene encoding the p53 protein. Its basic tasks in physiological conditions include maintaining genetic stability. It also plays an important role in eliminating abnormalities in the cell, by activating DNA repair mechanisms.^1–3^ It is a barrier against the initiation and progression of cancer. Its tasks include, among others: repairing genetic material^1,4^ regulating the cell cycle,^4^ participating in apoptosis^1,5^ and cell aging.^4^

Mutations in the TP53 gene cause poor prognosis in various types of cancer. These are usually somatic changes that occur in cells during carcinogenesis.^6^ The main effect of TP53 mutations in cancer cells is the loss of tumor suppressor properties, which requires therapeutic reactivation of the protein. All mutations occurring in the p53-dependent pathway can enable cancer development, and restoring normal p53 protein functions enables inhibition of tumor growth.^7^

In colorectal cancer, p53 exists as wild-type or mutant, guiding therapeutic approaches to prevent degradation or suppress dysfunction.^8^ Its activity is tightly regulated by interactions with proteins like MDM2, which negatively control its stability and function (Scheme 1).^9^ Blocking these negative regulators in tumors is crucial because MDM2 ubiquitinates p53, which leads to its degradation.^10^ Under normal conditions, MDM2 binds to p53, catalyzing its ubiquitination and degradation in the proteasome, which keeps p53 levels low and ensures proper cellular function.^11,12^ There is a negative feedback loop between MDM2 and p53—p53 activates the transcription of the MDM2 gene, and MDM2 limits the activity of p53, which helps maintain balance in the cell.^13–15^ In response to DNA damage, ATM/ATR kinases phosphorylate p53, preventing its interaction with MDM2 and stabilizing the protein. Stable p53 translocates to the nucleus, activating transcription of genes responsible for cell cycle arrest (e.g., p21), DNA repair (e.g., GADD45), and apoptosis (e.g., BAX).^16^ In situations of increased oncogenic activity or mitogenic signals (e.g., activation of c-Myc or Ras), ARF expression increases. AFR (Alternative Reading Frame) is a protein resulting from alternative opening of the reading frame within the p53 gene.^17^ ARF protein binds MDM2, preventing its interaction with p53, leading to p53 stabilization and induction of apoptosis in response to uncontrolled cell growth.^18^ MDM2 is also regulated by Akt kinase, which phosphorylates MDM2 in response to survival signals, promoting its transport to the nucleus and potentiating p53 inhibition. In this way, MDM2 balances between promoting cell survival and responding to stress.^18,19^ In cancers, MDM2 overexpression or TP53 mutations disrupt p53 function, driving uncontrolled proliferation and poor prognosis, especially in treatment-resistant tumors.^20^ This dysregulation promotes metastasis, therapy resistance, and genomic instability.^21^ Despite extensive research, no FDA-approved MDM2 inhibitors exist due to challenges in balancing efficacy and safety.^22,23^ Targeting the MDM2-p53 interaction remains a promising strategy to restore p53 function and inhibit tumor growth.^24^ The interplay between MDM2 and p53 in the regulation of apoptosis is summarized in Scheme 1.

**Scheme 1:**
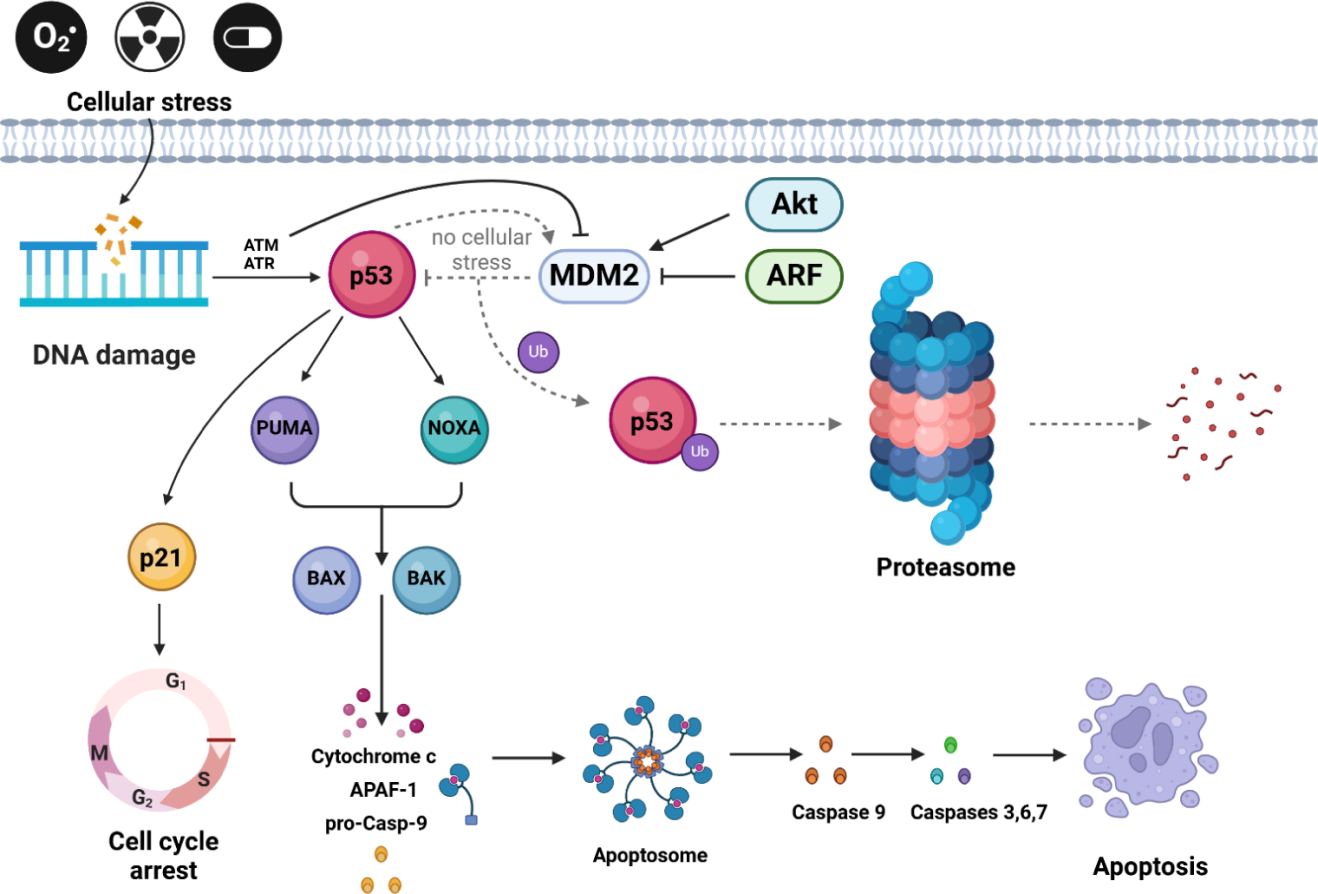
MDM2-Related Apoptosis Signaling Pathway^a^

a Created with BioRender.com.

The Bcl-2 protein inhibits apoptosis, playing a key role in carcinogenesis by promoting cancer cell survival and uncontrolled growth. Its interaction with p53 regulates apoptosis (Scheme 2).^25^ In the process of apoptosis, two main signaling pathways are distinguished: extrinsic and intrinsic (mitochondrial). The initiators of the intrinsic pathway are mutagenic factors such as UV radiation, cytotoxic drugs, or oxidative stress, which leads to DNA damage.^26^ DNA damage activates ATM and ATR, which stabilize p53, triggering the expression of proapoptotic genes while inhibiting survival-promoting ones. p53 inhibits BCL2, reducing its expression and facilitating the release of proapoptotic proteins from mitochondria.^27,28^ At the same time, p53 induces the expression of BH3-only proteins such as NOXA and PUMA.^25^ These proteins neutralize antiapoptotic proteins (e.g., BCL2), blocking their protective effect, and release proapoptotic proteins BAX and BAK, which become active. Active BAX and BAK oligomerize in the mitochondrial membrane, forming pores (MOMP, mitochondrial outer membrane permeabilization), which leads to the loss of mitochondrial membrane potential.^29^ As a result, proapoptotic proteins such as cytochrome c, Smac/DIABLO, and HtrA2/Omi are released from the mitochondrial intermembrane space. Cytochrome c initiates apoptosome formation, while Smac/DIABLO and HtrA2/Omi neutralize inhibitors of apoptosis (IAPs).^30–32^ Cytochrome c released into the cytoplasm binds to APAF-1 (Apoptotic Protease Activating Factor 1) and ATP/dATP, which induces conformational changes of APAF-1 and leads to its oligomerization, forming the apoptosome – a complex consisting of seven APAF1 molecules. The apoptosome recruits procaspase-9 via CARD domains (Caspase Recruitment Domain), which leads to its activation through mutual proteolytic cleavage. Active caspase-9 initiates the caspase cascade, activating effector procaspases such as caspase-3, caspase-6, and caspase-7.^33^ The caspase cascade leads to DNA fragmentation and cytoskeletal degradation, forming apoptotic bodies, which are phagocytosed to prevent inflammation.^34^ Overexpression of antiapoptotic Bcl-2 proteins hinders p53-dependent apoptosis, contributing to cancer cell resistance to stress-induced apoptosis and cytostatic drugs.^35,36^ The loss of p53 function and high Bcl-2 activity promote cancer progression and resistance to therapies like chemotherapy and radiotherapy.^10^ The mitochondrial pathway of apoptosis involving BCL2 regulation is depicted in Scheme 2.

**Scheme 2:**
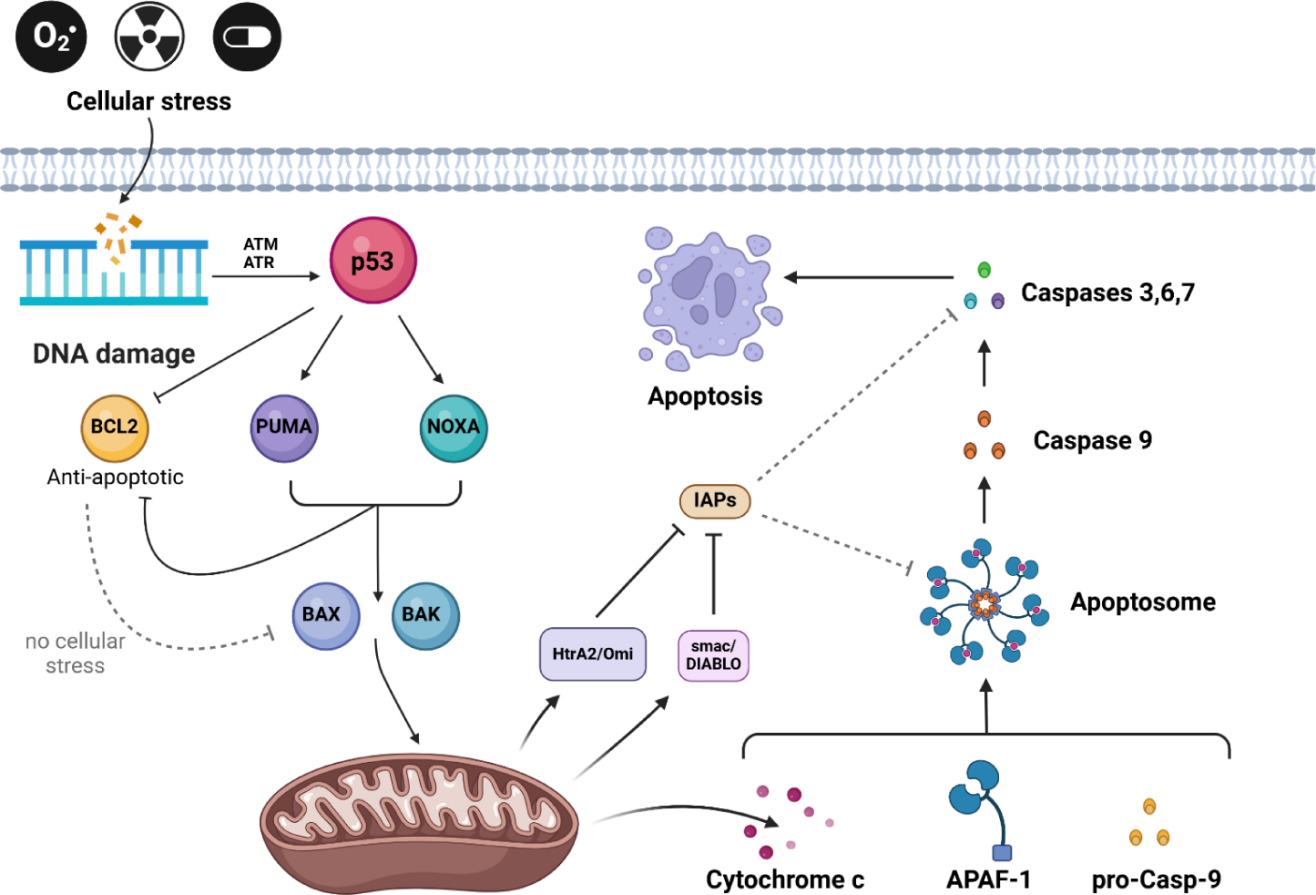
BCL2-Related Apoptosis Signaling Pathway[Fn sch2-fn2]

a Created with BioRender.com.

Apoptosis Inducing Factor Mitochondria Associated 2 (AIFM2), also known as Ferroptosis Suppressor Protein 1 (FSP1) or as PRG3 (p53-responsive gene 3), is homologous to AIF, which induces apoptosis independent of caspases.^37^ This mechanism involves mitochondrial dysfunction and pro-apoptotic factor release, particularly significant in cancers where classical apoptotic pathways are inhibited (Scheme 3).^38^ The exact mechanisms remain unknown, but it has been found that AIFM2 may perform this function by disrupting mitochondrial morphology and releasing proapoptotic factors.^38–41^ AIFM2 also acts as an oxidoreductase, protecting cells from ferroptosis—a form of cell death caused by oxidative stress, iron accumulation, and lipid peroxidation.^40,42–44^ AIFM2 reduces ubiquinone (CoQ10) to ubiquinol, an antioxidant stabilizing cell membranes against oxidative damage.^45^ This pathway is independent of glutathione and GPX4, making AIFM2 crucial in alternative ferroptosis protection.^46,47^ Its expression may be regulated by p53, which can inhibit AIFM2 during oxidative stress to promote ferroptosis and tumor cell elimination. Conversely, under metabolic stress, p53 may support AIFM2 to protect healthy cells.^48,49^ AIFM2 cooperates with mechanisms like the ESCRT-III complex, which repairs lipid peroxidation-induced membrane damage.^50,51^ In cancer cells with inactivated or mutated p53, AIFM2 may assume a protective role, which promotes cell survival and may contribute to therapy resistance. Blocking AIFM2, e.g., with specific inhibitors, is a potential therapeutic strategy that could increase the sensitivity of cancer cells to ferroptosis induction.^52^ In turn, increasing AIFM2 expression in cancer cells could contribute to the induction of cell death via apoptosis.^38^ Further studies on AIFM2 may reveal new therapeutic targets for cancer and diseases linked to oxidative stress. Modulating AIFM2 function could offer strategies for treating therapy-resistant cancers. The proposed mechanism of AIFM2-mediated apoptosis and ferroptosis regulation is illustrated in Scheme 3.

**Scheme 3:**
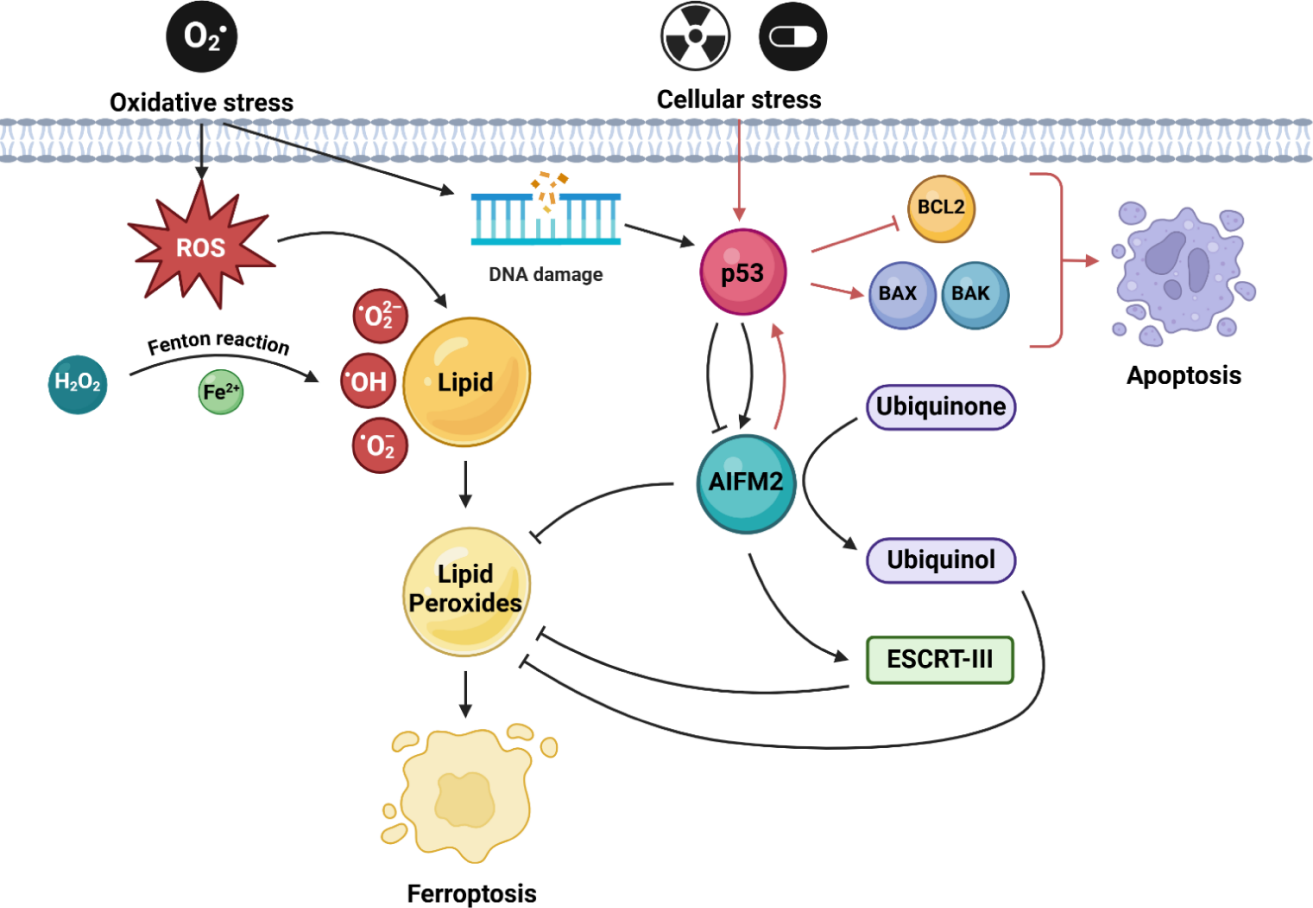
AIFM2-Related Apoptosis and Ferroptosis Signaling Pathway[Fn sch3-fn3]

a Created with BioRender.com.

RPL4 (Ribosomal Protein L4) is a key component of the ribosome that plays an important role in regulating the p53 pathway through interaction with the MDM2 protein. Studies have shown that RPL4 directly binds to MDM2, inhibiting its ability to ubiquitinate and degrade p53, which leads to the stabilization and activation of p53.^53^ Additionally, overexpression of RPL4 can lead to disorders in ribosome biogenesis, resulting in ribosomal stress and p53 activation. In turn, downregulation of RPL4 induces ribosomal stress, leading to p53 activation and cell cycle arrest.^54^ In the context of cancer, disorders in RPL4 expression can affect p53 function, which is important in the development and progression of cancer. Studies suggest that understanding the role of RPL4 in regulating p53 could lead to new therapeutic strategies for treating cancer.^54^ Here, the related gene of the ribosomal protein RPL41, from the 60S subunit, was taken as a housekeeping and reference gene followed by RT-qPCR reactions.

Mutations in the p53 protein promote cancer progression. In this way, the activity of the mutated p53 protein is also changed in relation to the wild-type p53 protein. Therefore, the p53 protein has become an ideal therapeutic target.^55^ The common occurrence of mutations in the TP53 gene found in cancers has prompted research on the p53 protein. Currently, they mainly include restoring the proper functioning, stabilization or degradation of the faulty protein.^6,9^ Unfortunately, the development of such a drug is difficult due to the many possible mutations occurring in the TP53 gene. Despite this, research on therapy based on the p53 protein has been developing very quickly in recent years.

Phenothiazines are a class of chemical compounds that have long been used in medicine, mainly as antipsychotics and antihistamines.^56^ Recent studies suggest that some phenothiazines may have, in addition to other properties, significant anticancer activity.^57–61^ Their mechanism of action following occurrence may be due to interaction with p53, which plays a key role in secondary regulation and apoptosis. Phenothiazines may stimulate apoptosis in cancer cells by activating p53-dependent pathways. Phenothiazines can affect the interaction between p53 and its negative regulator MDM2. MDM2 binds to p53, leading to its degradation. Phenothiazine analogues can block this interaction, increasing p53 stability and promoting its antitumor functions.^62^ Structural modifications to these compounds may lead to changes in their biological properties, which may be crucial to increasing their effectiveness as potential anticancer drugs.^63–65^

One strategy for modifying the phenothiazine structure, in addition to introducing new substituents to the thiazine nitrogen atom, is the introduction of various mono- or bicyclic aromatic or heteroaromatic rings in place of the benzene ring. Our long-standing research on modifying the phenothiazine system has relied on the modification of phenothiazines by introducing pyridine, quinoline, and naphthalene rings in place of the benzene rings (Figure 1). As a result of such modifications, we have obtained a variety of aza- and diazaphenothiazines with the structures of dipyridothiazines, diquinothiazines, quinobenzothiazines, and naphthoquinothiazines. Some of these compounds have demonstrated promising immunosuppressive and anticancer activity against human cancer cell lines derived from the colon, breast, kidney, lung, ovary, prostate, central nervous system, melanoma, and leukemia.^64,65^

**Figure 1:**
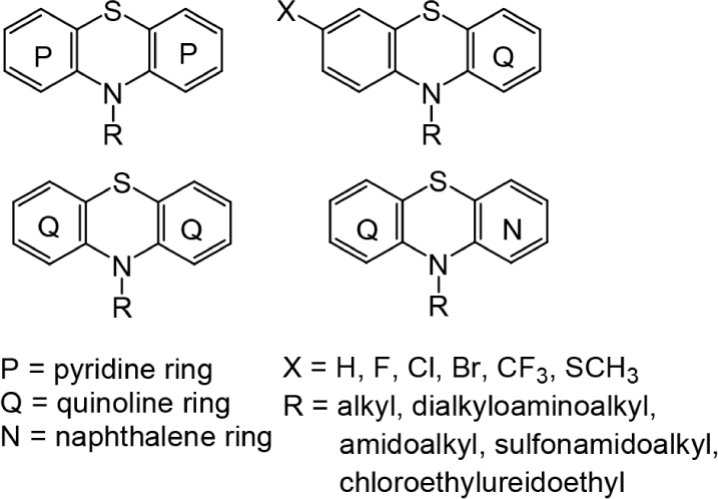
Structures of pyridine-, quinoline-, and quinoline-naphthalene-modified phenothiazines.

The tested substances belong to the structural analogues of phenothiazines, in which benzene rings were replaced by azine systems (pyridines – **BM1, BM2** and quinoline – **MJ1, MJ2**). Compounds **BM1** and **BM2** are dipyridothiazines, modified with pyridine rings, whose correct chemical name is 10H-1,9-diazaphenothiazine­(10H-dipyrido­[3,2-b:2“,3′-e]­[1,4]­thiazine **BM1**) and 10-propargyl-1,9-diazaphenothiazine­(10-(prop-2-yn-1-yl)-10H-dipyrido­[3,2-b:2”,3′-e]­[1,4]­thiazine **BM2**) (Figure 2).^66^ The structure of the compound **BM1** was first described in a US patent in 1957. Unfortunately, the information in the report was very general and did not contain precise biological aspects. At present, this compound is effectively obtained in the sulfuration reaction of dipyridylamine using a microwave reactor. The structure of this molecule was confirmed by NMR, MS, and X-ray techniques. The **BM2** compound was obtained in the alkylation reaction of 10H-1,9-diazaphenothiazine using propargyl bromide. Similarly to the **BM1** molecule, its structure was confirmed spectroscopically. Antitumor activities of the **BM1** and **BM2** compounds were tested. **BM1** was a very active substance against the C-32 melanoma line with the inhibitory concentration (IC) parameter (IC_50_ = 3.83 μM). On the other hand, the BM2 derivative showed high activity against the SNB-19 glioma line (IC_50_ = 3.85 μM) and C-32 melanoma (IC_50_ = 3.37 μM). Preliminary TP53 gene expression analysis studies on **BM1** and **BM2** compounds indicated that these molecules had the ability to disrupt gene expression in SNB-19, C-32, and MDA-MB-231 cells.^66^

**Figure 2:**
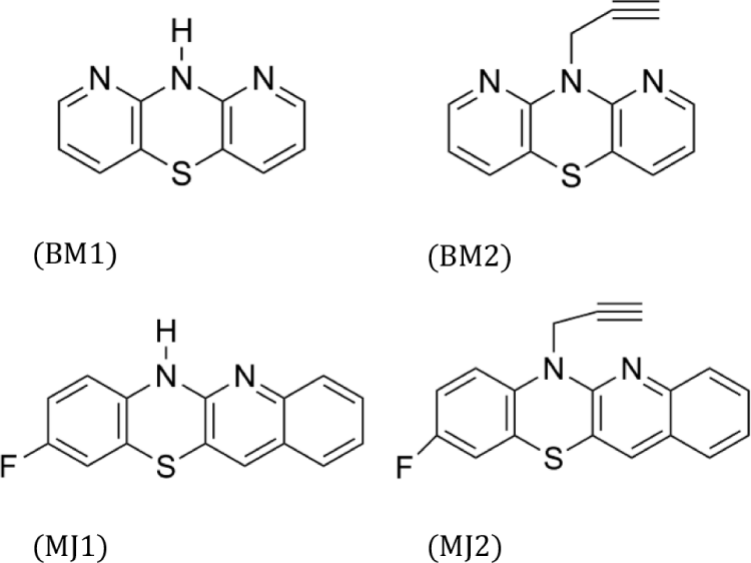
Structures of tested compounds: BM1, BM2, MJ1, MJ2.

The next two compounds are tetracyclic phenothiazines in which one of the benzene rings has been replaced by a quinoline system, and the benzene ring has a fluorine substituent (Figure 2). The **MJ1** compound (6H-9-fluoroquino­[3,2-*b*]­benzo­[1,4]­thiazine) was obtained by annulation of 2,2′-dichloro-3,3′-diquinolyl disulfide with p-fluoroaniline.^67^ The **MJ2** derivative (9-fluoro-6-propargyl-quino­[3,2-*b*]­benzo­[1,4]­thiazine) was obtained by alkynylation of the **MJ1** compound using propargyl bromide.^68^ The cytotoxic and immunomodulatory activity of the **MJ2** molecule has been described in the literature.^68^ It also showed activity against the following cancer lines with the growth inhibition (GI) parameter: lymphoma L1210 (GI50 = 2.28 μg/mL), colon: SW948 (GI50 = 44.50 μg/mL), CX-1 (GI50 = 10.83 μg/mL), and skin cancer A-431 (GI50 = 2.84 μg/mL) Table 1.

**Table 1:** Compounds Used in the Experiment with Their IC_50_ Values

Chemical compound symbol	IC_50_ [μM] HCT116+/+	IC_50_ [μM] HCT116–/–
MJ1	56.31 ± 5.62	80.78 ± 4.33
MJ2	36.37 ± 4.30	52.82 ± 6.37
BM1	197.25 ± 9.88	261.90 ± 7.98
BM2	82.89 ± 5.97	87.13 ± 3.45

The compounds for this study were selected due to the analogy in the structure of the substituents at the thiazine nitrogen atom, as well as promising preliminary results for antitumor activity in *in vitro* studies. Critical to the study of phenothiazines is their potential interaction with p53, a key regulator of cell cycle and apoptosis. Strutural changes in phenothiazines can affect the activation of p53-dependent pathways, such as BCL2 and MDM2 gene expression, making these compounds promising candidates for the treatment of cancers with wild-type p53.^64^ At the same time, other modifications may be more effective in cancers with p53 mutations, opening up new therapeutic possibilities.^69,70^ Phenothiazine derivatives with modifications at the 10-position of the ring, similarly to compounds BM1 and BM2, were tested for their ability to generate reactive oxygen species (ROS), which may lead to the induction of p53-dependent apoptosis.^69^ In turn, MJ1 and MJ2 are distinguished by the presence of propargyl and halogen substituents, which, according to studies, may increase their affinity for DNA repair proteins and modulate the activity of protein kinases involved in the p53 pathway.^71^ The choice of these four compounds was also dictated by their diverse lipophilicity and solubility profiles, which allowed for the assessment of the influence of physicochemical parameters on their biological activity. Often, the limitation of phenothiazines is their low solubility, which affects bioavailability and requires the use of higher concentrations, increasing the risk of adverse effects.^72^ This problem highlights the need for further research on improving the pharmacological properties of phenothiazines, such as changing lipophilicity or introducing hydrophilic systems, which may influence their biological activity. The present study aimed to investigate how these diverse chemical features influence the activation or inhibition of the p53 pathway in cancer cells. The images presented in Tables 2–5 depict the results of incubation with the compounds at a concentration of 100 μM, where precipitation was observed. However, for the MTT assay, appropriate dilutions of the compounds were used, effectively eliminating precipitation while ensuring accurate determination of IC_50_ values.

**Table 2:**
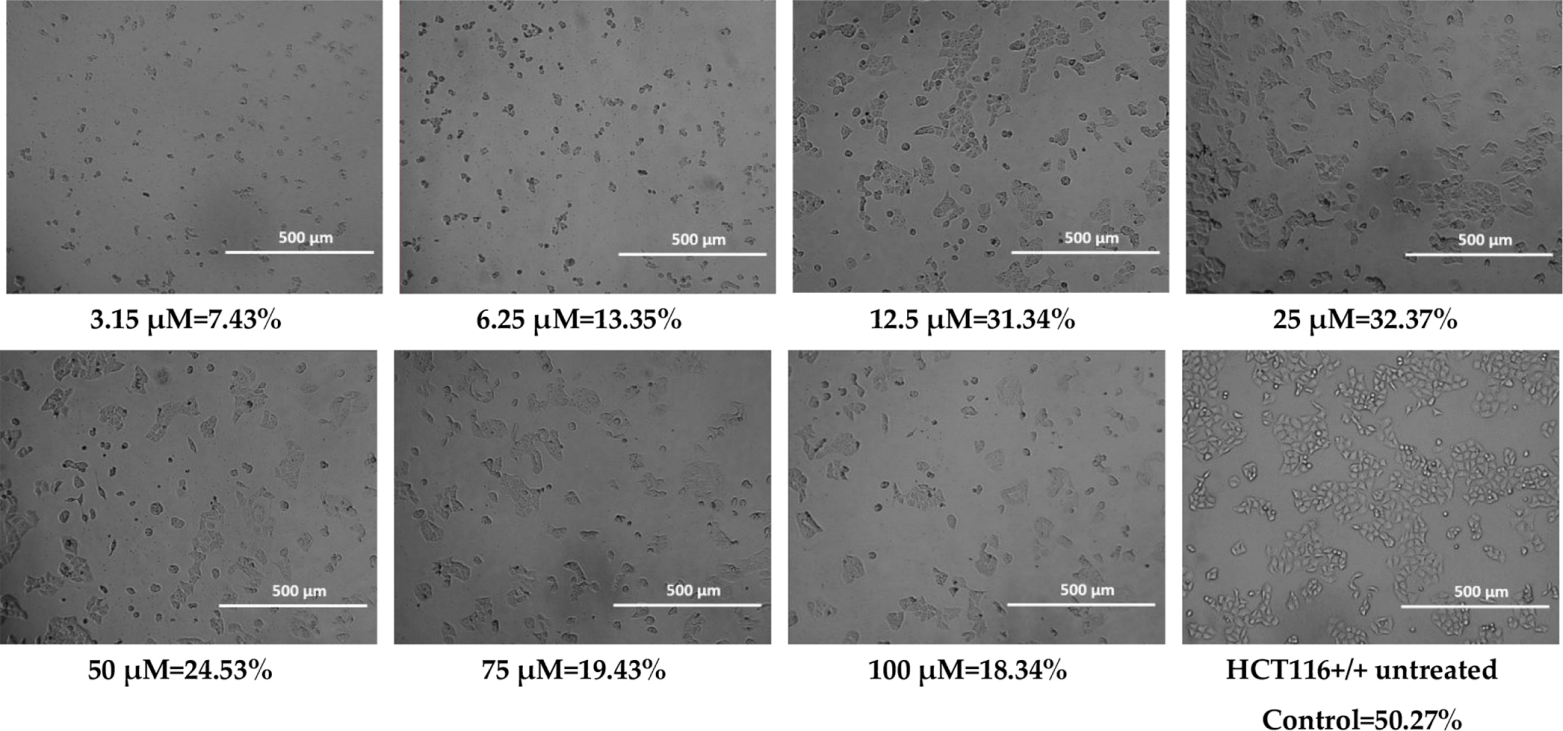
Cell Confluence of HCT116 Cell Line for Compound BM1

**3:**
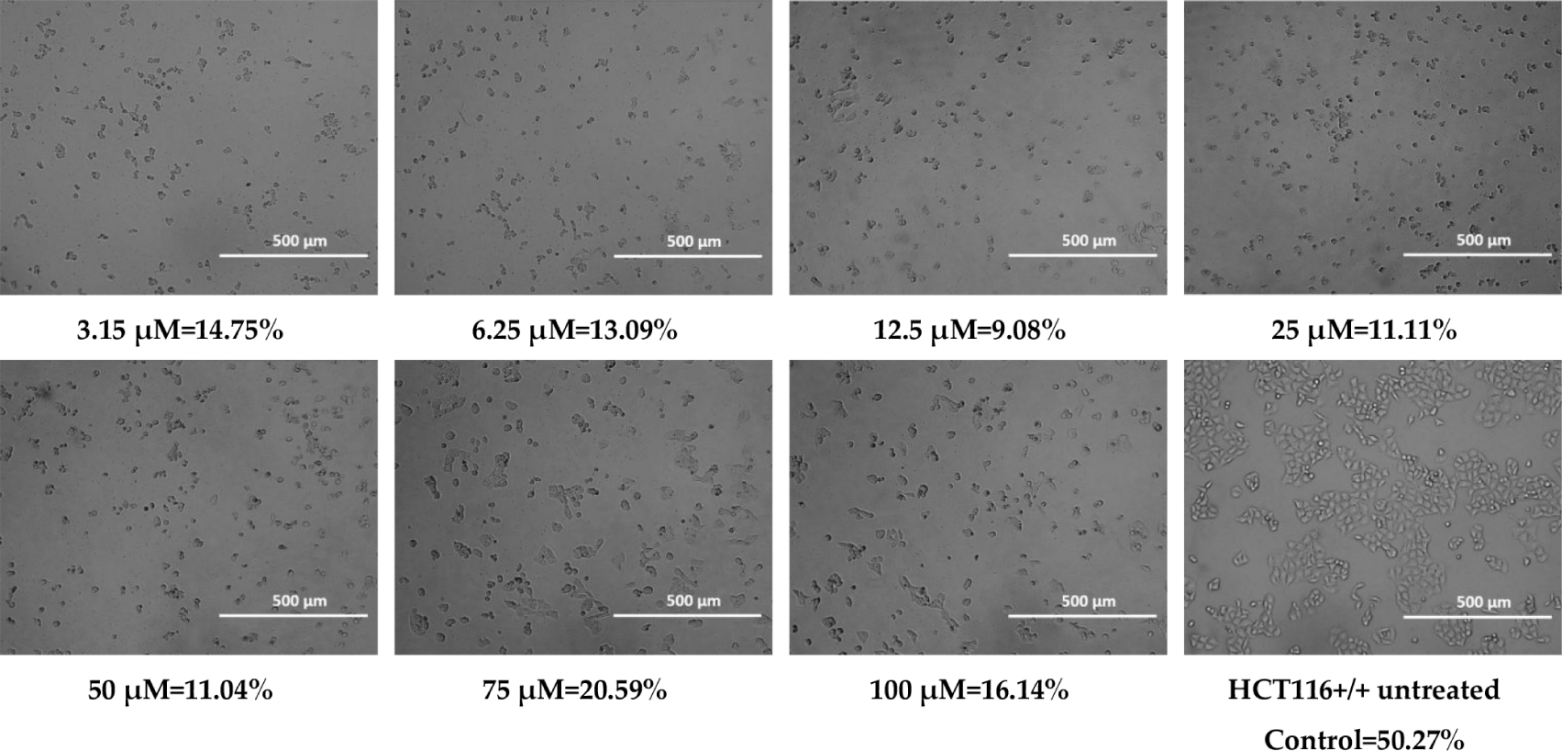
Cell Confluence of HCT116 Cell Line for Compound BM2

**4:**
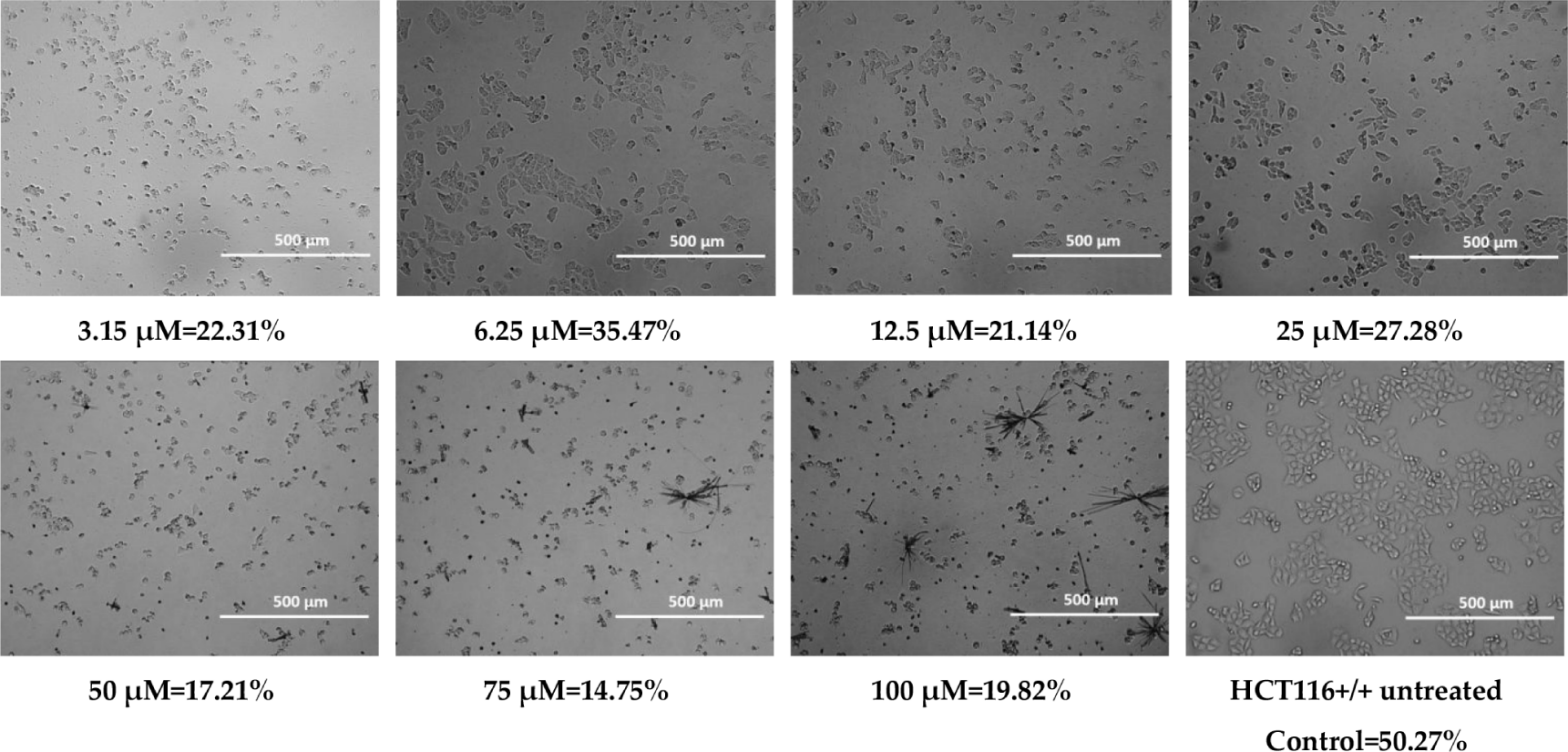
Cell Confluence of HCT116 Cell Line for Compound MJ1

**Table 5:**
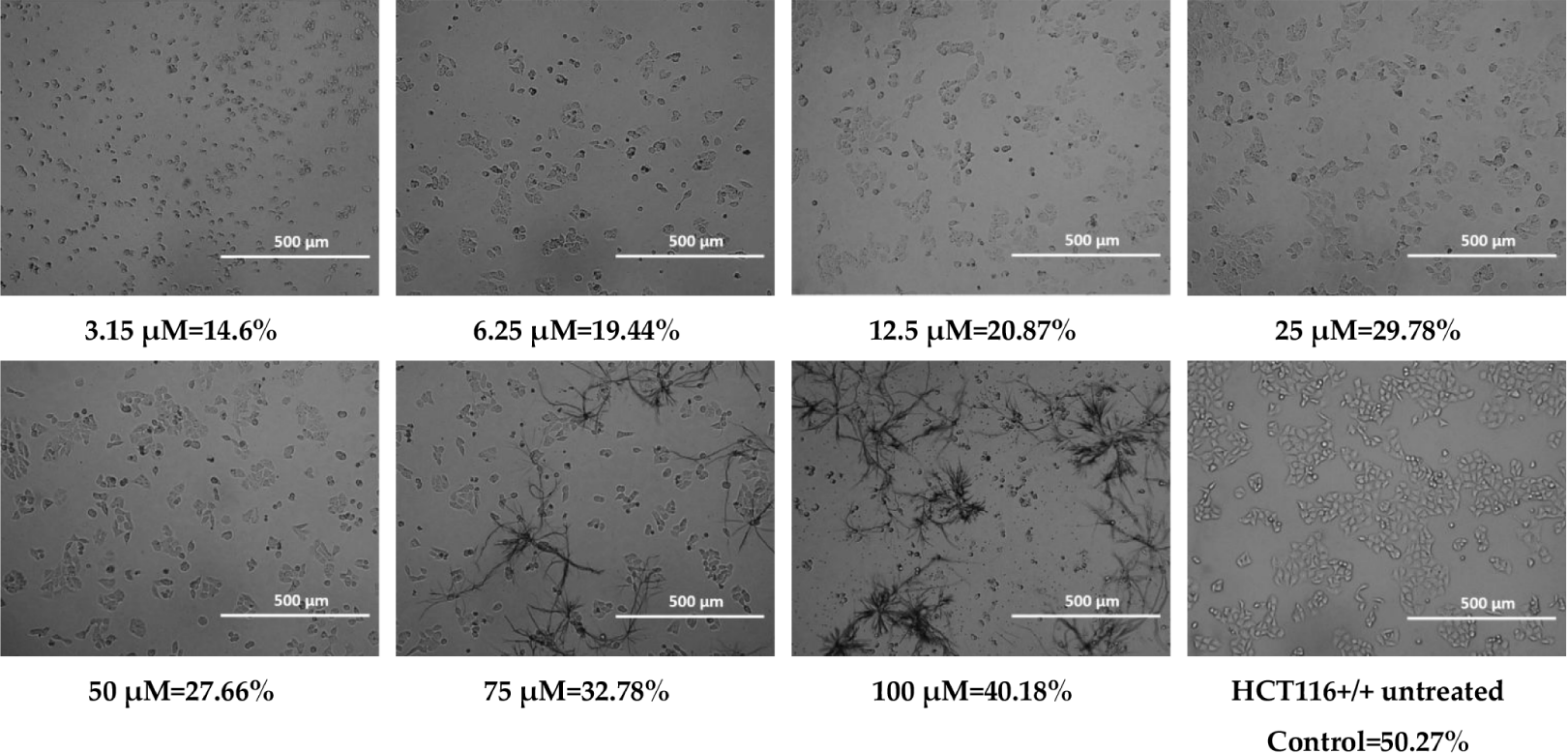
Cell Confluence of HCT116 Cell Line for Compound MJ2

Understanding the role of p53 protein in carcinogenesis and the possible influence of phenothiazines on its regulators has been considered important for the development of medicine. The aim of this work was to investigate the influence of selected structural analogues of inhibitors of p53-dependent signaling pathways.

## 2 Results

The study began with the MTT test to determine the IC_50_ parameter values for the tested phenothiazines, and then microscopic observations and real-time polymerase chain reaction (RT-PCR) were performed.

### 2.1 MTT Colorimetric Test

The cytotoxicity of selected chemical compounds, **BM1**, **BM2**, **MJ1**, and **MJ2**, was analyzed using a colorimetric assay. The HCT116 cell lines with wild-type p53 protein status, HCT116 with mutated p53 protein were used for the experiment. Incubations of cells with compounds were carried out for two time periods: 24 and 72 h. The IC_50_ values were determined based on averaged cell viability data, representing the half-maximal concentration required to inhibit cell growth. The obtained results are presented in Table 1.

Based on the performed MTT colorimetric tests, IC_50_ values were calculated for chemical compounds designated as **BM1**, **BM2**, **MJ1**, and **MJ2**. The IC_50_ value is the concentration of the compound required to inhibit cell viability and proliferation by 50% compared to the control, which was not exposed to the compound. Of all the tested compounds, **MJ2** showed the lowest IC_50_ values in all tested cell lines. Despite the lowest values, the IC_50_ remains high. In turn, the **BM1** compound had the least favorable IC_50_ values. The MTT tests performed show that the tested chemical compounds are not the best cytostatics for selected cancer lines, because high concentrations are required to inhibit cell growth. Such doses pose a risk of low safety of using these compounds against healthy cells. Despite this, the tested compounds are characterized by cytotoxicity for cancer cells, and the obtained IC_50_ values were used for further experiments.

### 2.2 Confluency

Cell confluency was evaluated after 72 h of treatment with the test compounds. Representative images and quantitative data for HCT116 cells treated with 100 μM of **MJ1**, **MJ2**, **BM1**, and **BM2** are presented below.

During the observations, morphological changes were observed in cancer cells treated with the tested substances, such as cell shrinkage, cytoplasmic thickening, and cell separation from the rest, which indicates cell death. The observed changes may suggest the occurrence of apoptosis, or programmed cell death.^73^ At higher concentrations of **MJ1** (50 μM, 75 μM, and 100 μM) and **MJ2** (75 μM and 100 μM), precipitation of the compounds was observed, which may have led to an overestimation of confluency due to false-positive signal detection.

### 2.3 Real-Time Polymerase Chain Reaction (RT-PCR)

The key experiment performed was the real-time polymerase chain reaction. The studies were conducted for the reference gene RPL41 and the genes studied: AIFM2, BCL2, and MDM2. Two HCT116 lines were used to compare the effect of p53 protein status on the relative increase in expression.

#### 2.3.1 Experiment 1

RT-PCR was performed on the HCT116 cancer line with a wild-type p53 protein status (HCT116+/+) and HCT116 with a mutated p53 protein (HCT116–/−). The reference RPL41 gene and the p53 gene were selected for experiment 1. Samples incubated for 24 h were selected as a control sample, and samples after 72-h incubation were selected as the test sample. After calculating the relative expression level for both cell lines, a high expression level for the p53 gene was demonstrated in the HCT116 line with a wild-type p53 protein status (Chart [Fig chart1]).

**1 chart1:**
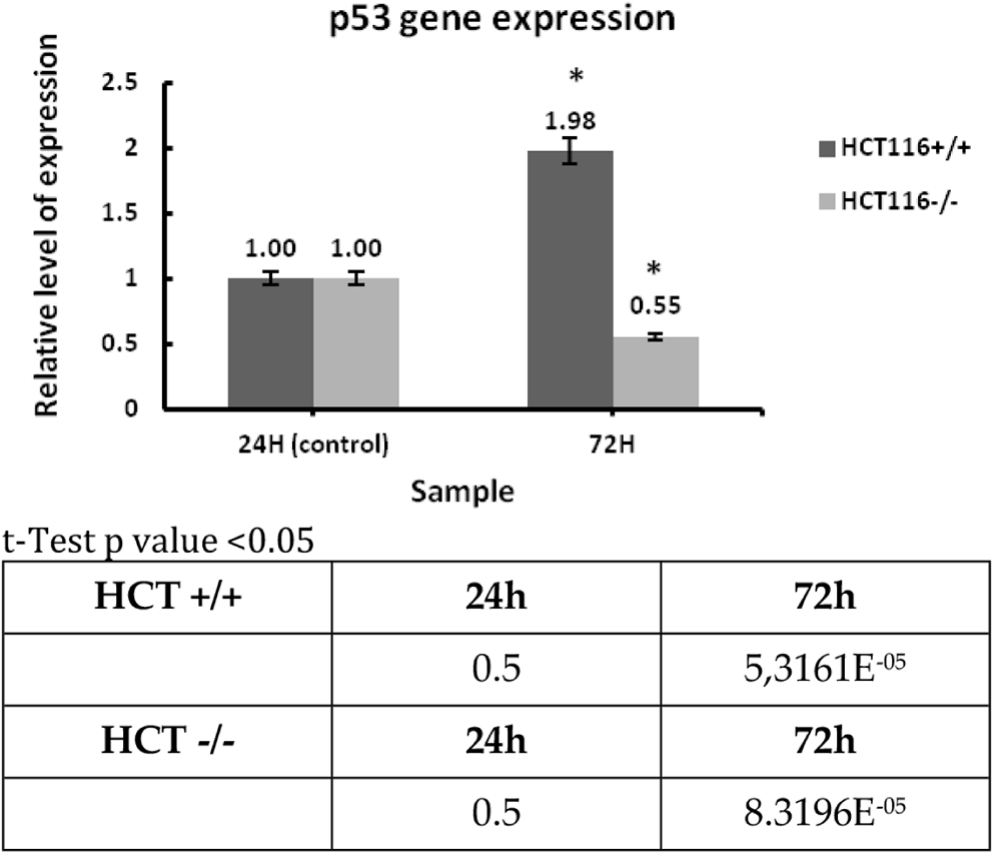
Relative Expression Level of p53 Gene in HCT116+/+ and HCT116–/– Cell Lines

Results are presented as mean ± SD, calculated from three experiments and compared to the untreated controls at the 24 h time point. Statistical significance was calculated using the t-test where *p* < 0.05 and is indicated by an asterisk (*); bolded in the table below.

#### 2.3.2 Experiment 2

RT-PCR concerned the HCT116 cell line with wild-type p53 protein status and HCT116 with mutated p53 protein. The reference gene RPL41 and the tested genes were used for the experiment. The studies were performed for the compounds **MJ1**, **MJ2** and **BM1**, **BM2** (administered in IC_50_ doses), after 24 h of incubation.

##### 2.3.2.1 AIFM2

RT-PCR was performed on the HCT116 cell line with wild-type p53 protein status and HCT116 with mutated p53 protein. The reference gene RPL41 and the tested AIFM2 were used for the experiment. The studies were performed for the compounds **MJ1**, **MJ2** and **BM1**, **BM2** (administered in IC_50_ doses), after 24 h of incubation (Chart [Fig chart2]).

**2 chart2:**
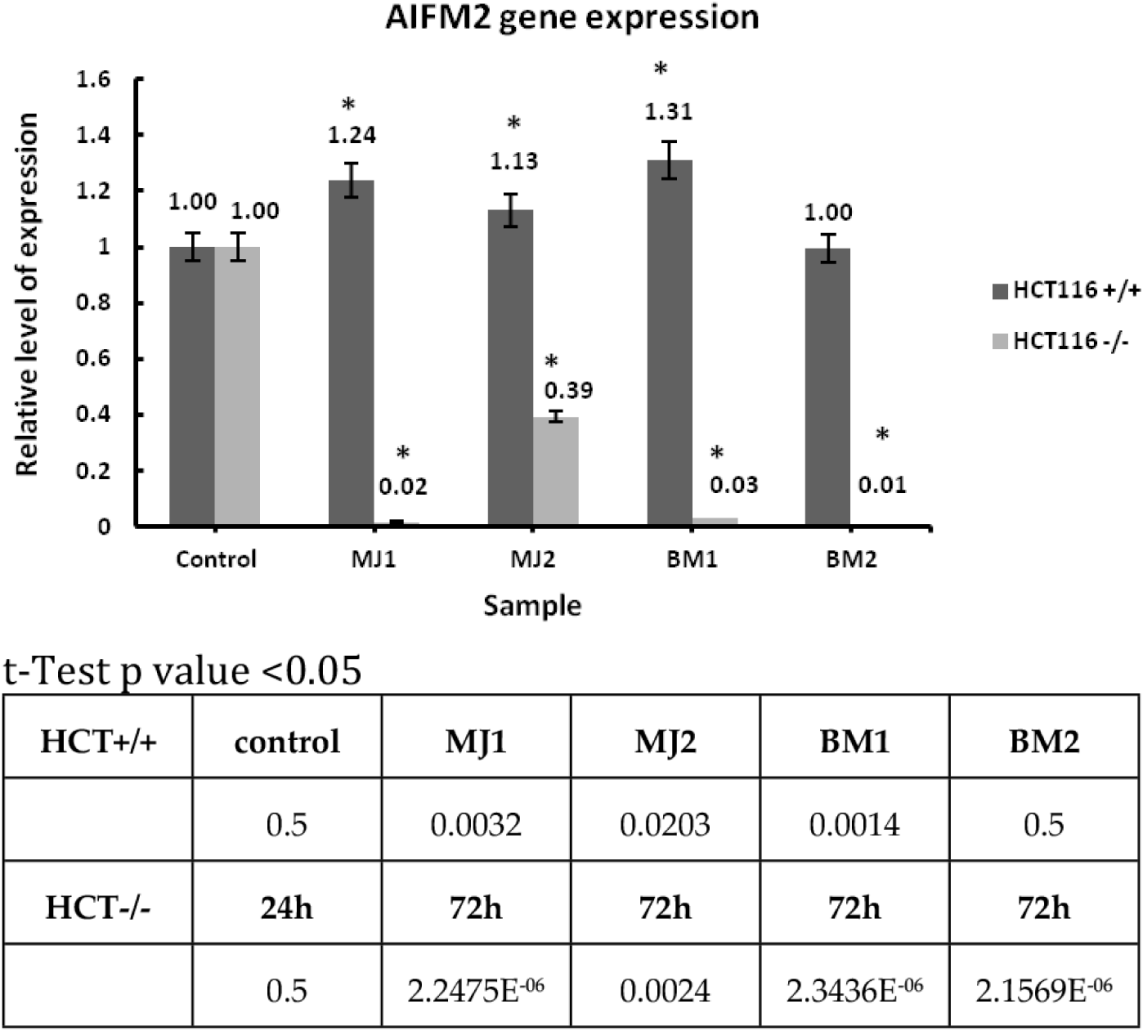
Relative Expression Level of the AIFM2 Gene for Compounds MJ1, MJ2, BM1, BM2 (with a Concentration Value Equal to IC_50_) and 24 h Incubation

Results are presented as mean ± SD, calculated from three experiments and compared to the untreated controls. Statistical significance was calculated using the t-test where *p* < 0.05 and is indicated by an asterisk (*); bolded in the table below.

For experiment 2, AIFM2 was used as a test gene, which encodes an apoptosis-inducing factor associated with canonical activation of the mitochondrial death pathway.^74^ For the line with mutant p53 protein, all compounds resulted in silencing of AIFM2 expression. In comparison to the line with wild-type p53 protein, the relative expression level increased.

##### 2.3.2.2 BCL2

Experiment 3 RT-PCR was performed for the HCT116 cell line with wild-type p53 status and HCT116 with mutated p53. The reference gene RPL41 and BCL2 were used for experiment 3 (Chart [Fig chart3]). BCL2 acts as a regulator of apoptosis for the p53-dependent signaling pathway.^75^

**3 chart3:**
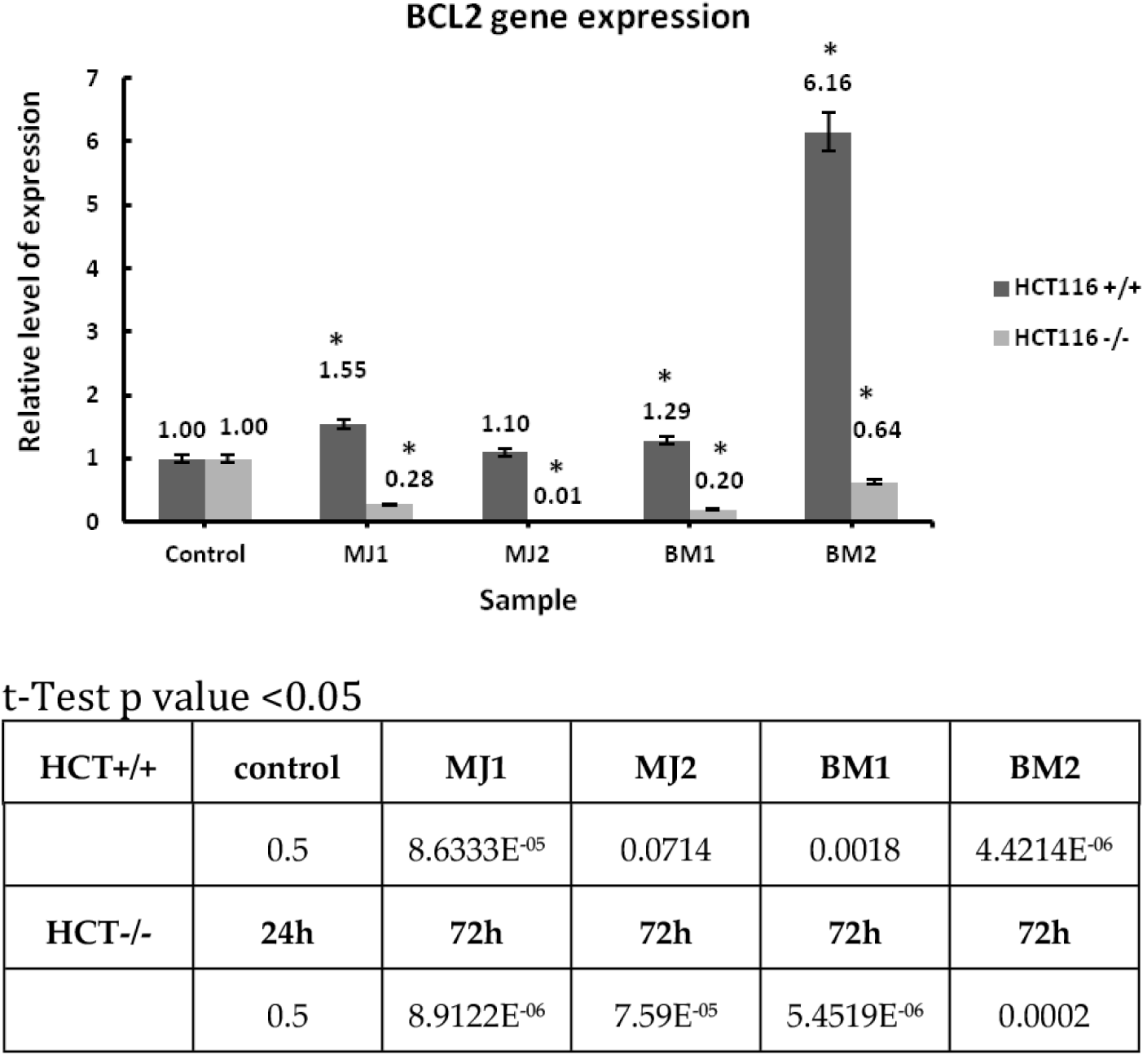
Relative Expression Level of the BCL2 Gene for Compounds MJ1, MJ2, BM1, BM2 (with a Concentration Value Equal to IC_50_) and 24 h Incubation

Results are presented as mean ± SD, calculated from three experiments and compared to the untreated controls. Statistical significance was calculated using the t-test where *p* < 0.05 and is indicated by an asterisk (*); bolded in the table below.

For the experiment in which the expression level of the BCL2 gene, which acts as a regulator of apoptosis, was measured, a decrease in expression was observed for compounds **MJ1**, **MJ2**, **BM1**, and **BM2** in the case of the HCT116 line with mutated p53 protein. The highest relative increase in gene expression was obtained for the **BM2** compound of the HCT116 line with wild-type p53 protein status. For the remaining compounds, the expression level was maintained within the control level. This means that the HCT116 line with mutated p53 protein has a lower expression level of BCL2 in contrast to the line with wild-type p53 protein.

##### 2.3.2.3 MDM2

RT-PCR was performed for the HCT116 cell line with wild-type p53 protein status and with mutated p53 protein. The reference gene RPL41 and the studied gene MDM2 were used for the experiment (Chart [Fig chart4]).

**4 chart4:**
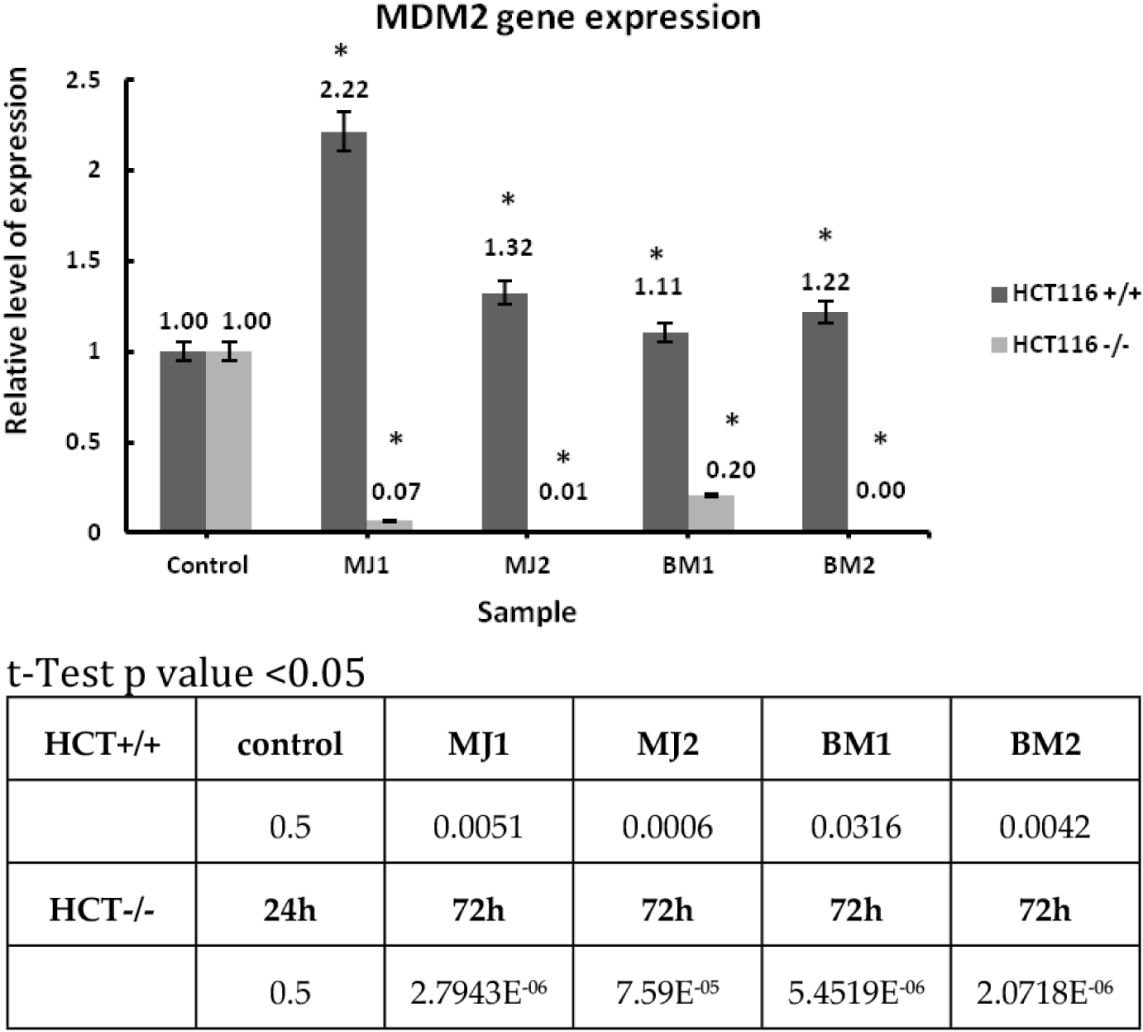
Relative Expression Level of the MDM2 Gene for Compounds MJ1, MJ2, BM1, BM2 (with a Concentration Value Equal to IC_50_) and 24 h Incubation

Results are presented as mean ± SD, calculated from three experiments and compared to the untreated controls. Statistical significance was calculated using the t-test where *p* < 0.05 and is indicated by an asterisk (*); bolded in the table below.

MDM2 is also known as E3 ubiquitin-protein ligase.^76^ For the line with wild-type p53 protein, a clear increase in the relative expression level is visible after incubation with the **MJ1** compound, and comparable values for the remaining compounds, compared to the control. For the cancer line with mutated p53 protein, the expression level is muted for all tested compounds.

## 3 Discussion

The development of medicine and pharmacology forces scientists to create new, safe and effective drugs whose mechanisms of action are well understood. In order to avoid undesirable consequences of using drugs and various side effects, many different tests are performed to check their safety. Cell cultures come to the rescue, which are relatively cheap, simple and easy to control, in contrast to in vivo animal or clinical studies. They provide information on the cytotoxicity and biological activity of drugs. The cytotoxic test, which is the MTT test, allows for the assessment of the cytotoxicity of selected chemical compounds, their ability to affect the viability, growth, and damage of cells. Compounds that have tested and confirmed cytotoxicity are able to inhibit proliferation and the formation of cell colonies, stop the cell cycle and DNA replication.^77^ Two cell lines were selected for the experiments: HCT116 cell lines with wild-type p53 protein status and HCT116 with mutated p53. Both lines produce the p53 protein, and additionally lines with a mutated protein variant were selected. It was crucial to select these lines in order to learn about the p53-dependent cellular signaling pathways and the impact of the tested chemotherapeutics on them.

The first anticancer activity of phenothiazines was described in the 1980s. In later years, structures were modified by substituting various substituents in position 10, which resulted in their anticancer properties. Modifications of phenothiazines via the pyridine ring led to the creation of dipyridothiazines. The key aspect for compounds **BM1** and **BM2**, which made them compounds with very good anticancer properties, is the presence of a dipyridothiazine scaffold and the arrangement of two azine nitrogens in tricyclic ring systems. Compound **BM2**, a derivative of compound **BM1** with a substituted propynyl group, is a highly active compound against glioma lines SNB-19 and melanoma C-32. According to literature studies, the efficacy of compound **BM2** was confirmed for reducing the expression of the TP53 gene. For the glioblastoma multiforme cell line, the induction of mitochondrial apoptosis was observed based on the BAX/BCL2 gene expression ratio. On the melanoma line, however, this compound affected cell death via the intrinsic mitochondrial apoptosis pathway.^64,66,78^ The **MJ1** and **MJ2** compounds are very valuable compounds with high anticancer activity. The modifications of the **MJ2** compound were aimed at reducing cytotoxicity by substituting the propargyl group in position 6, while maintaining strong antiproliferative properties.^64,67,68^

The first test performed was the colorimetric MTT test. During the MTT tests, it was important to maintain the same experimental conditions for all replicates. The concentrations of the tested compounds, the time of cell exposure to the drug, the density of cell seeding, the time of the experiment, sterile conditions in the culture and other factors played a very key role in obtaining the reliability and consistency of the results. Another important aspect was the selection of the appropriate concentration range for the tested compounds.^79^ The MTT test is an easy and safe test, characterized by high repeatability. Based on the colorimetric MTT tests, the IC_50_ values were calculated for the tested chemical compounds, marked with the symbols **BM1**, **BM2**, **MJ1**, **MJ2**. The IC_**50**_ value means the concentration of the compound that is able to inhibit cell viability and proliferation by 50% compared to the untreated control. The best IC_50_ values for all lines are found for the **MJ2** compound. For the HCT116 line with wild-type p53 protein IC_50_ = 36.37 μM and IC_50_ = 57.82 μM for HCT116 with mutated p53 protein. Despite obtaining the lowest values, IC_50_ are still relatively high. The worst activities, i.e., concentrations with the highest effective values, are possessed by compound **BM1** for the HCT116 line with mutated p53 protein, IC_50_ = 261.90 μM, for the HCT116 line with wild-type p53 protein IC_50_ = 197.25 μM. The **MJ1** compound for the HCT116 line with wild-type protein has IC_50_ = 56.31 μM, for the HCT116 line with mutated protein IC_50_ = 80.78 μM. On the other hand, the **BM2** compound for the HCT116 line with wild-type p53 protein status IC_50_ = 82.89 μM and for the HCT116 line with mutated p53 protein IC_50_ = 87.13. The MTT test conducted allows us to conclude that for selected cancer lines, the tested chemical compounds are not the best cytostatic. The concentrations of compounds that must be administered to inhibit cell growth are very high. The obtained IC_50_ results, combined with the morphological observation of dead cells, may indicate that the apoptotic processes induced by phenothiazines may be delayed; therefore, the IC_50_ may be relatively high, indicating the need for longer contact with the drug to achieve the full cytotoxic effect.

When assessing the confluence using a JuLI FL Live fluorescence microscope from NanoEntek, the selected HCT116 cell line showed the precipitation of the compound for the **MJ1** compound at concentrations of 50 μM, 75 μM, and 100 μM, and for the **MJ2** compound at concentrations of 75 μM and 100 μM. This could have been caused by the high concentration of the compounds. The pH of the culture could also have had an adverse effect. Long-term cell culture (conducted for 24–72 h) has an acidic pH, which could have affected the precipitation of the compounds over time, especially at higher concentrations. However, for all tested compounds, the confluence was significantly lower compared to the control. Morphological assessment also revealed changes between cells not treated with the tested compounds and cell lines after the use of the tested analogues, which confirms the cytostatic effect of phenothiazines (Tables 2–5). Despite the high IC_50_ values obtained, the morphological changes suggest the induction of cell death already at lower values (3.15 μM) of all tested compounds. The discrepancy between the IC_50_ values obtained in the MTT assay and the changes observed microscopically may be related to the limitations of the MTT assay, which measures only metabolic activity, not taking into account early morphological changes indicative of cell death. The results suggest that the morphological effects of phenothiazines on cells appear already at sublethal concentrations, which may be related to the activation of apoptotic mechanisms or cellular stress before the overall metabolic activity is significantly reduced.

The key experiment performed was the real-time polymerase chain reaction. The studies were conducted for the reference gene RPL41 and the genes studied: AIFM2, BCL2, and MDM2. Two HCT116 lines were used to compare the effect of p53 protein status on the relative increase in expression. For the purpose of summary, a Table 6 was created showing the inhibition or lack of inhibition of expression for selected genes, based on the genes expression from Charts [Fig chart1]–[Fig chart4].

**Table 6:** Expression Level Results for the Studied Genes and Tested Compounds Divided into HCT116 Cell Lines with Wild-Type p53 Protein Status and with Mutant p53 Protein; Based on Genes Expression from Charts [Fig chart1]–[Fig chart4] ^a^,^b^

Cell line	Tested gene			
	P53			
HCT116 p53+/+	↑			
HCT116 p53–/–	↓			
	AIFM2			
	MJ1	MJ2	BM1	BM2
HCT116 p53+/+	↑	↑	↑	↑
HCT116 p53–/–	↓	↓	↓	↓
	BCL2			
	MJ1	MJ2	BM1	BM2
HCT116 p53+/+	↑	↑	↑	↑
HCT116 p53–/–	↓	↓	↓	↓
	MDM2			
	MJ1	MJ2	BM1	BM2
HCT116 p53+/+	↑	↑	↑	↑
HCT116 p53-/-	↓	↓	↓	↓

a (↓) Downregulation—decreasing of gene expression.

b (↑) Upregulation—increasing of gene expression.

AIFM2 plays a key role as a regulator of apoptosis in the p53-dependent signaling pathway. This association underscores the importance of apoptosis as one of the primary mechanisms by which the tumor suppressor p53 exerts its effects. Importantly, AIFM2 is tightly bound to p53 and its expression is directly regulated by this protein. As a result, apoptosis-inducing factor 2 plays a significant role in tumor development, underscoring its importance in cancer biology.^80^ For the HCT116 tumor line with the status of wild-type p53 protein and the tested AIFM2 gene, the relative expression level for all tested compounds was higher than the control sample. The interaction of p53 with AIFM2 may be important in this case. For HCT116 lines with mutated p53 protein, the expression for all compounds was low, which suggests that the expression was silenced. BCL2 as the next gene tested belongs to the group of protooncogenes. Mutated p53 protein has very strong links with BCL2. Strong genetic, chemical and signaling connections between them are of fundamental importance for oncology. Interacting proteins play a key role in the process of carcinogenesis and drug response. BCL2 is mainly responsible for the carcinogenesis of hematological malignancies.^25^ During the RT-PCR experiment for the HCT116 line with mutated p53 protein and the **MJ1** and **MJ2** compounds, an obvious silencing of the expression level was observed, which means that the tested compounds inhibited the expression of this gene. The compounds tested turned out to be effective inhibitors. For the same cancer line, slightly worse results, but still lower than the control sample, were obtained by the **BM1** and **BM2** compounds. These results confirm the reduction of BCL2 gene expression by selected compound, which was confirmed in previous studies.^64^ For the HCT116 line with wild-type p53 protein, the expression level was higher for all tested compounds than for the control sample. This means that the compounds did not inhibit BCL2 expression with the participation of wild-type p53 protein. The MDM2 gene was chosen for the last experiment. This is another gene that strongly interacts with the p53 protein. Unfortunately, regardless of the p53 protein status, MDM2 can cause various damages and delays in repair. For the HCT116 cell line with wild-type protein, the relative expression level for all tested compounds did not decrease. All results are higher than the control sample. However, for the line with the mutated p53 protein, the expression level decreased for all compounds. For the **MJ2** and **BM2** compounds, the expression is completely silenced. This means that these compounds inhibited the expression of the MDM2 gene, which affects the regulation of the p53 protein.

## 4 Materials and Methods

### 4.1 Chemical Synthesis

The tested dipyridothiazines BM1, BM2 ^66^ and quinobenzothiazines MJ1 ^67^ and MJ2 ^68^ were synthesized and characteristed according to previously described literature procedures.

### 4.2 Cell Culture Management

The experiments utilized well-established HCT116 cell lines, which included two types: one with functional p53 and the other without p53. The HCT116 cell line lacking the TP53 gene, created through a biallelic knockout method^81^ was kindly provided by Dr. B. Vogelstein. The HCT116 cell line is a human colon cancer cell line isolated from an adult male tumor. The HCT116 in vitro cell line is an excellent tool for studying the mechanisms of tumor growth and metastasis. It is also useful in assessing the effects of drugs. The model of this line very well mimics the properties and structure of solid tumor cells. During the experiments, studies were conducted on the tumor line:

• HCT116 with wild-type p53 protein status (HCT116 p53+/+);

• HCT116 with mutated p53 protein (HCT116 p53–/−).

A vessel with selected cell lines (HCT116 p53 wild type, HCT116 with mutated p53), was taken from the cell bank. Cells were thawed at room temperature. Using an automatic pipette, the contents of the vessel were transferred to a 15 mL falcon and suspended in 3 mL of fresh DMEM culture medium. Then, centrifugation for 3 min (2000 rpm). After centrifugation was completed, the old medium was removed from above the cells and 3 mL of fresh medium was added again. The obtained cell suspension was transferred to a new, appropriately labeled culture bottle.

The selected cell lines were cultured in plastic culture bottles selected appropriately depending on the type of cell line. The bottles were stored in specially designated incubators with an appropriate constant temperature of 37 °C and a CO_2_ concentration of 5%. The humidity inside the incubator was maintained at about 80%, which was intended to prevent the cultures from drying out. DMEM culture medium was used for all studies.

### 4.3 Confluency

Cell confluency was monitored using the JuLI FL Live fluorescence microscope (NanoEntek), which enables real-time imaging and automatic confluency calculation based on brightfield images. For each experiment, 3 × 10^5^ HCT116 cells were seeded per well in 6-well plates and incubated under standard conditions (37 °C, 5% CO_2_). After cell attachment, the cells were treated with 100 μM of **MJ1**, **MJ2**, **BM1**, or **BM2** and incubated for 72 hours.

At the end of the incubation period, images were acquired using the JuLI FL system and confluency was automatically calculated using the integrated software.

### 4.4 MTT Test

The MTT test is classified as a biochemical test. It is used to assess the cytotoxicity of selected chemical compounds on cells. This test is based on the ability of mitochondrial dehydrogenase to convert a tetrazolium salt to a water-insoluble formazan. The amount of the reduced MTT reagent is directly proportional to the oxidative activity of mitochondria. Therefore, this test indicates cell viability (metabolic activity). The product of this reaction is insoluble in water, which is why organic solvents such as DMSO or acidic isopropanol are used.

The IC_50_ (inhibitory concentration) coefficient is used to express cytotoxic activity. This is the concentration at which cell viability or proliferation is inhibited by 50%, in relation to the control sample.

In order to perform the MTT colorimetric assay, 96-well plates were first prepared on which selected cell lines were seeded in the amount of 10 thousand per well. For this purpose, old medium was poured out from selected culture bottles containing HCT116 lines with wild-type p53 protein status and HCT 116 with mutated p53 protein. 3 mL of PBS was added, which was used to rinse the bottom of the bottle and poured out. Then, 3 mL of trypsin was added to the adherent lines and placed in incubation for 3–4 min. After the specified time, the bottle was removed from incubation and the condition of the cells was checked under a microscope. A double volume of culture medium was added to neutralize trypsin. The whole was carefully pipetted. Twice 10 μL of the mixture was taken and applied to the prepared Bürker chamber from both sides. The Bürker chamber was placed under a microscope and the sum of cells from the appropriate squares was counted. After determining the number of cells in the suspension, it was supplemented with culture medium according to the calculations. In the next step, 100 μL of culture medium solution with selected cell lines was added to the prepared 96-well plate. The cells were incubated for 24 h at 37 °C.

Chemical compounds (**BM1**, **BM2**, **MJ1**, **MJ2**) were dissolved in 1 mL of DMSO. After adding DMSO, the whole was shaken until dissolved for about 10 min. To improve solubility and ensure complete dissolution, the solutions were subjected to brief sonication in a water bath sonicator for 10 min at room temperature. Then new falcons were prepared and volumes of compounds corresponding to 0.1 mM were added and DMEM medium was supplemented to 5 mL. After 24 h, the medium was removed from all wells of the incubated 96-well plates. Then, the solutions of chemical compounds (**BM1**, **BM2**, **MJ1**, **MJ2**) were added in appropriate amounts. The concentrations of the tested compounds were in the dilution ranges from 3.15 μM to 100 μM. The experiment was set up in two time periods: 24 and 72 h at 37 °C. After the time had elapsed, the entire culture medium was collected from all plates. 50 μL of MTT reagent (concentration 0.5 mg/mL) ((3-(4,5-dimethyl-thiazol-2-yl)-2,5-diphenyltetrazolium bromide) was added to each well and incubated for 2–3 h. Then the MTT reagent was collected and 75 μL of acidic isopropanol was added. The whole was pipetted and placed on a thermomixer for 10 min. After the incubation was completed, the absorbance level was measured using a spectrophotometer at a wavelength of 570 nm using an Epoch plate reader. IC_50_ values were calculated based on three independent biological experiments, each performed in triplicate. Mean IC_50_ values were determined using linear interpolation, and the results are presented as mean ± standard deviation (SD).

### 4.5 RT-PCR

The real-time PCR method is also known as real-time polymerase chain reaction. The PCR diagnostic method is one of the most commonly used methods to identify and replicate a selected nucleic acid fragment.^67,68^ For the polymerase chain reaction, culture plates were prepared on which HCT 116 cell lines with wild-type p53 protein status and HCT 116 with mutated p53 protein were incubated. The culture was carried out in two time periods: 24 and 72 h at 37 °C. Control cultures and experimental cultures were prepared, to which compounds at concentrations equal to the IC_50_ value were added. After the appropriate incubation time, the entire medium was collected from the cell cultures into the falcons. The plates were washed with 1 mL of PBS and transferred to the falcons. Then, 1 mL of trypsin was added to detach the cells from the bottom and they were placed for 2–3 min for incubation. Neutralization was carried out with the contents of the falcon and everything was placed back into it. The falcon was placed in a centrifuge and spun for 3 min (2000 rpm). After centrifugation, all supernatant medium was removed and 400 μL of phenosol was added under a fume hood using filter tips. The whole was carefully pipetted for cell lysis. Samples were frozen at −20 °C, where they were stored until further experiment and RNA isolation.

#### 4.5.1 RNA Isolation

RNA isolation began by thawing previously collected samples. RNA isolation was performed according to the instructions included with the A&A Biotechnology Total RNA isolation kit (Total RNA mini, A&A biotechnology). The experiment began by incubating the samples for 5 min at 50 °C. 200 μL of chloroform, which was not included in the kit, was added to the lysate. The samples were gently mixed by inverting the tubes several times and left for 3 min at room temperature. Then they were centrifuged for 10 min at 12,000 rpm. In the next step, the upper fraction was collected in a fume hood and placed in new tubes, and 250 μL of isopropanol was added. The whole was mixed thoroughly and transferred to the minicolumns included in the kit. The minicolumns were placed in a centrifuge and centrifuged for 1 min at 12,000 rpm. After centrifugation, the minicolumns were transferred to new 2 mL tubes and 700 μL of A1 wash solution, which is included in the total RNA isolation kit, was added. Then, it was centrifuged again for 1 min at 12,000 rpm. The minicolumns were removed from the tubes and the filtrate was poured out. 200 μL of A1 wash solution was added again and centrifuged for 2 min at 12,000 rpm. The columns were placed in new clean 1.5 mL tubes with appropriate labeling. 50 μL of sterile water, included in the kit, was added to the bed at the bottom of the minicolumn. The samples were left for 3 min at room temperature and then centrifuged for 1 min at 12,000 rpm. In the last step, the minicolumns were removed and the obtained RNA was stored at −80 °C until further experiment.

#### 4.5.2 Reverse Transcription

In order to perform reverse transcription, the RNA concentration obtained during isolation was measured using a NanoDrop 2000 spectrophotometer. For this purpose, 1.5 μL of material was taken from the samples and placed in the device. After measuring the RNA concentration, the maximum amount of cDNA that could be obtained from the sample with the lowest concentration was calculated. Then, the amount of sterile water needed for the experiment was calculated. After completing all calculations, the reaction solution was prepared according to the instructions and the assumed number of samples used in the experiment.

The reaction solution contains:

• 5x NG cDNA Buffer – 4 μL,

• 50 μM Oligo­(dT)­20 – 1 μL,

• NG DART RT Mix – 1 μL,

• Sterile water (RNase-free water).

In the next step, appropriate amounts of sterile water and RNA (total 14 μL) were added to the tubes. Then, the reaction mixture (6 μL) was added, which had been prepared earlier. The whole was vortexed and centrifuged. In the last step, the tubes were placed in a thermocycler. According to the manufacturer’s protocol, they were incubated for 1 h at 50 °C, and then incubated for another 5 min at 85 °C to terminate the reaction. In this way, cDNA was obtained. After incubation, the samples were placed in a freezer (−20 °C) and stored until further analysis.

#### 4.5.3 Real-Time PCR

At the beginning of the experiment, the samples prepared during reverse transcription (cDNA) were thawed on ice. Then, based on the concentrations obtained during reverse transcription, the values needed to perform the RT-PCR reaction were calculated so that 1 ng of cDNA was available per reaction (well). The experiment was divided into four parts. Two experiments are presented as an example (Experiment 1 and 2).

Experiment 1 - conducted on a control culture. Two sets of primers were used for control RT-PCR: for the p53 gene and the reference RPL41 gene. Volumes of cDNA, Mastermix, and sterile water for two genes for experiment 1:

cDNA (5,2 μL)

MASTERMIX (60 μL)

STERILE WATER (30,8 μL)

P53 (12 μL)

RPL41 (12 μL)

Experiment 2 - conducted on culture exposed to the test compounds, BM1. BM2, MJ1, and MJ2. For this purpose, two sets of primers were used: for the AIFM2/BCL2/MDM2 gene and the reference gene RPL41. Volumes of cDNA, Mastermix and sterile water for two genes for experiment 2:

cDNA (4.2 μL)

MASTERMIX (60 μL)

STERILE WATER (31.8 μL)

AIFM2/BCL2/MDM2 (12 μL)

RPL41 (12 μL)

Each reaction was started by mixing cDNA, sterile water and Mastermix. The whole was vortexed and centrifuged. The samples were divided into 2 Eppendorfs, 48 μL each. Each sample was for one pair of primers. Then the appropriate primers were added in a volume of 12 μL. After adding the sample, it was vortexed and centrifuged again. In the next step, an RT-PCR plate with the appropriate number of wells was prepared. Twenty μL of the mixture was carefully applied to each well. After the entire application, the RT-PCR plate was carefully sealed and centrifuged for 3 min at 3000 rpm. After centrifugation, the plate was placed in a thermal cycler. The experiment was carried out according to the protocol (Brilliant III Ultra-Fast SYBR Green QPCR Master Mix, Quick Reference Guide for the Bio-Rad CFX96 Real-Time PCR Detection System).

Reaction profiles and results for both the tested and reference genes were performed using Bio-Rad CFX Maestro 1.1 software from Bio-Rad. Analysis and calculation of the results obtained during RT–PCR were performed using Microsoft Excel. For the obtained Cq (Ct) threshold results, the relative expression level was calculated. The test sample contained cancer cells incubated with the tested compounds, and the control sample contained only cells incubated in the medium. The *R* = 2−ΔΔCt method was used for calculations. This method allows for the calculation of the relative expression level (R). It is based on the fluorescence intensity of the selected gene at the Cq cutoff point.^82^

$$R=\frac{2\Delta \mathrm{Cq}\;\mathrm{tested}}{2\Delta \mathrm{Cq}\;\mathrm{reference}}$$

R – relative level of gene expression,

ΔCq tested – difference in threshold values (Cq) for the tested gene,

ΔCq reference – difference in threshold values (Cq) for the reference gene.

For selected genes used during the real-time PCR experiment, Table 7 was prepared, which contains the primer sequences BCL2, MDM2, AIFM2, and RPL41.

**Table 7:** Primer Sequences Used in the RT-QPCR Reaction

Gene	Primer forward	Primer reversed
BCL2	CTTCAGGGACGGGGTGAAC	GGATCCAGGTGTGCAGGTG
MDM2	GCCCTTCGTGAGAATTGGCT	CCTCAACACATGACTCTCTGG
AIFM2	CTGCCCTTCTCTCATCTTATCCT	CTGCCTCACCATGTCCTCATAG
RPL41	TCCTGCGTTGGGATTCCGTG	ACGGTGCAACAAGCTAGCGG

### 4.6 Statistical Analysis

All biological experiments were repeated at least three times, with three technical repetitions each. The data were shown as means ± standard error (S.E) of three assays. Student’s t-test was applied, and *p* < 0.05 value was considered as statistically significant. The statistical significance is indicated by star (*) on the Charts [Fig chart1]–[Fig chart4], and the changes are compared to the untreated controls, respectively. For the calculations the MS office version 2010 of Excel was used.

## 5 Conclusions

The aim of the work was to investigate the effect of selected structural analogues on inhibitors of p53 protein-dependent signaling pathways. Several tests were performed during the experimental studies. The first study was the MTT colorimetric test. It allowed calculating the IC_50_ values for the tested compounds **MJ1**, **MJ2**, **BM1**, **BM2**. The results obtained during the test were not satisfactory compared to the literature values. Despite high IC_50_ values, morphological changes indicate induction of cell death already at lower concentrations (3.15 μM). This discrepancy is due to the limitations of the MTT assay, which does not take into account early morphological changes. The results suggest that phenothiazines induce apoptotic effects or cellular stress already at a sublethal stage, before metabolic activity decreases.

The key experiment was the real-time polymerase chain reaction. These experiments aimed to determine the relative expression level of selected genes on cell lines containing two different p53 protein statuses. This allowed determining the effect of the tested compounds on selected lines and genes. The results confirm the effect of the p53 protein status on the level of gene expression.

The conclusions from the conducted experiment indicate a significant relationship between the status of the p53 protein and the response of HCT116 cells to phenothiazines. It was observed that in cells with a functional, wild-type p53 protein, treatment with phenothiazines led to increased expression of the AIFM2, BCL2, and MDM2 genes, which are involved in the mechanisms of apoptosis and control of genome stability. On the other hand, in cells with mutated p53, phenothiazines caused the opposite effect, reducing the expression of these genes. These results suggest that the activity of the p53 protein plays a key role in modulating the cellular response to phenothiazines. In cells with wild-type p53, the expression of AIFM2, BCL2, and MDM2 increased in response to phenothiazines, which may indicate the activation of caspase-independent apoptosis mechanisms, in which p53 plays a regulatory role. In turn, in cells with mutated p53, reduced expression of these genes after treatment with phenothiazines may indicate a reduced ability to activate apoptotic pathways, which may contribute to resistance to treatment.

The reduced response in cells with mutated p53 may also suggest that p53-dependent molecular mechanisms are important for the full activity of phenothiazines in inducing cell death. Furthermore, the need to use high concentrations of phenothiazines to induce cytotoxic effects may indicate a potential need for further optimization of these compounds in the context of cancer therapy, especially in cases where p53 is mutated or defective.

The conclusions from the conducted studies indicate that the chemical structure of the phenothiazines studied plays a key role in their interaction with the DNA of cancer cells.^83^ These phenothiazines exhibit the ability to intercalate into the DNA structure, which leads to disruption of its integrity and activation of appropriate DNA repair mechanisms and apoptotic pathways.^84^

Fluoro- and propargyl groups increase the lipophilicity of these compounds, which facilitates their penetration into the cells. In addition, the presence of a propargyl group may affect DNA repair enzymes and apoptosis mechanisms, contributing to the selectivity of phenothiazines.

The relationship between p53 status and cellular response to phenothiazines suggests that these compounds modify the expression of genes involved in apoptosis, such as AIFM2, BCL2, and MDM2, depending on the presence or mutation of p53. In cells with wild-type p53, phenothiazines increased the expression of these genes, suggesting the activation of caspase-independent apoptosis mechanisms, whereas in cells with mutant p53, the expression of these genes was reduced, indicating a reduced response to DNA damage.^62^

In summary, the anticancer and antiproliferative properties of the tested compounds were confirmed. These findings indicate that phenothiazines may be a promising element of future therapeutic strategies, especially in cancers with mutations in the p53 gene. Understanding their action in the context of p53 protein status opens new possibilities in cancer treatment, which may contribute to the development of more precise and effective anticancer therapies, taking into account the specific genetic status of cancer cells. Future studies should include canonical phenothiazines as controls to further delineate structure–activity relationships and reinforce specificity.

## Supplementary Material



## Notes

Detailed synthetic procedures and characterization data (^1^H/^13^C NMR, MS) for **BM1**, **BM2**, **MJ1**, and **MJ2** (PDF)

## Abbreviations

AIF: apoptosis-inducing factor
AIFM2: apoptosis-inducing factor mitochondrion-associated 2
Akt: Protein kinase B (PKB)
APAF-1: apoptotic protease activating factor 1
ARF: p14ARF alternative reading frame
ATM: ataxia-telangiectasia mutated
ATR: ATM- and Rad3-related
BAK: Bcl-2 antagonist killer 1
BAX: Bcl-2-associated X protein
BCL2: B-cell CLL/lymphoma 2
BH3-only: interacting-domain death agonist
CARD: caspase recruitment domain
c-Myc: cellular myelocytomatosis oncogene
CoQ10: ubiquinone
ESCRT-III: endosomal sorting complex required for transport-III
FDA: Food and Drug Administration
GADD45: growth arrest and DNA damage
GPX4: (glutathione peroxidase 4)
HtrA2/Omi: high temperature requirement protein A2 (HtrA2) Omi stress-regulated endoprotease
IAPs: Inhibitors of Apoptosis
MOMP: mitochondrial outer membrane permeabilization
NOXA: Phorbol-12-myristate-13-acetate-induced protein 1
Pro-Casp-9: procaspase-9
PUMA: p53 upregulated modulator of apoptosis
ROS: reactive oxygen species
Smac/DIABLO: second mitochondria-derived activator of caspase/direct inhibitor of apoptosis-binding protein with low pI
Ub: ubiquitin
