# Supplementary material for: The Interaction of Structural Analogues of Phenothiazines in p53-Dependent Cellular Signaling Pathways
Source: ACS Omega. 2026 Jan 2;11(1):371–87. doi: 10.1021/acsomega.5c04145 (PMC12809562; doi:10.1021/acsomega.5c04145)
Supplement: Supplementary file 1 [file ao5c04145_si_001.pdf]

Supporting Information

The interaction of structural analogues of phenothiazines in p53-dependent cellular signaling pathways

Klaudia Giercuszkiewicz-Haśnik[a,b,c]*, Paulina Pawicka[a], Małgorzata Jeleń[d], Beata Morak-Młodawska[d] and Magdalena Skonieczna[a,b]*

[a] Department of Systems Biology and Engineering, Silesian University of Technology, Akademicka Street 16, 44–100 Gliwice, Poland;

[b] Centre of Biotechnology, Silesian University of Technology, Krzywoustego Street 8, 44-100 Gliwice, Poland;

[c] Faculty of Medical Sciences in Katowice, Medical University of Silesia, Medyków Street 18, 40-752 Katowice, Poland;

[d] Department of Organic Chemistry, Faculty of Pharmaceutical Sciences in Sosnowiec, Medical University of Silesia in Katowice, Jagiellońska Street 4, 41-200 Sosnowiec, Poland;

* Corresponding author's email: klaudia.giercuszkiewicz@polsl.pl; magdalena.skonieczna@polsl.pl

Scheme S1 Synthesis of 10*H*-1,9-diazaphenothiazine (10*H*-dipyrido[3,2-b:2',3'-e][1,4]thiazine **BM1**) and 10-propargyl-1,9-diazaphenothiazines (10-(prop-2-yn-1-yl)-10H-dipyrido[3,2-b:2',3'-e][1,4]-thiazine **BM2**)

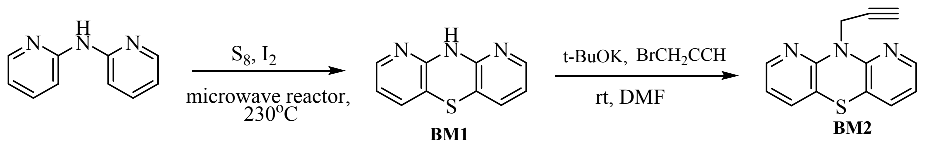

Synthesis performed in a microwave reactor. A mixture of 2,2`-dipyridinylamine (0.171 g, 1 mmol), elemental sulphur (0.064 g, 2 mmol) and small crystal of iodine was added to the clean quartz reactor vessel. The mixture was heated at 230 °C during 30 min. The obtained brown oil was purified by column chromatography (aluminium oxide,$CHCl_3$) to give 10*H*-1,9-diazaphenothiazine (**BM1**) (0.085 g, 43%) mp 157-158 °C, ^{1}H NMR ($CDCl_3$) δ: 6,74 (dd, *J* = 7.8 Hz), 7.16 (dd, *J* = 7,8 Hz, *J* = 1.8 Hz), 7.96 (dd, *J* = 4.8 Hz; *J* = 1.8 Hz), 8.33 (s, 1H, N-H). ^{13}C NMR ($CDCl_3$): 112.86 (C4a, C5a), 118.61 (C3,C7), 133.53 (C4 ,C6), 145.91 (C2,C8), 152.35 (C9a, C10a), EI MS m/z: 201 (M, 100). HR MS (EI) m/z for: [$C_{10}H_7N_3S$ + H] calc. 202.0439. Found: 202.0455 [1].

In a round-bottomed flask, in dry (10 ml) DMF, 80mg (0.72 mmol) potassium tert-butoxide was added to a suspension of 10*H*-1,9-diazaphenothiazine (**BM1**) (0,100 g, 0.5 mmol). The mixture was stirred at room temperature for 1 h. Then to the solution was added drop-wise a solution of propargyl bromide (0.080 g, 0.64 mmol) in dry toluene. The solution stirred at room temperature 24 h and poured into water (20 ml), extracted with methylene chloride (20 ml), dried with Na_2SO_4, evaporated to the beige oil. The residue was purified by column chromatography (silica gel, $CHCl_3$) to yield 10-propargyl-1,9-diazaphenothiazine (**BM2**) (0.085 g, 71%); mp. 119-120 °C, ^{1}H NMR: δ: 2.17 (s, 1H, CH), 5.07 (s, 2H, CH_2), 6.84 (dd, *J* = 7.5 Hz, *J* = 5.1 Hz 2H, H_3, H_7), 7.28 (dd, *J* = 7.8 Hz, *J* = 1.8 Hz; 2H, H_4, H_6), 8.12 (dd, *J* = 4.8 Hz; *J* = 1.8 Hz, 2H, H_2, H_8). EI MS: 239 (M, 90), 200 (M-CH_2CCH, 100). HR MS (EI) m/z for: [$C_{13}H_9N_3S$ + 1] calc. 240.0595 Found: 240.0599 [1].

Scheme S2 Synthesis of 6*H*-9-fluoroquinobenzothiazine (6*H*-9-fluoroquino[3,2-b]benzo[1,4]thiazine **MJ1**) and 9-fluoro-6-propargyl-quinobenzothiazine (9-fluoro-6-propargyl-quino[3,2-b]benzo[1,4]-thiazine **MJ2**)

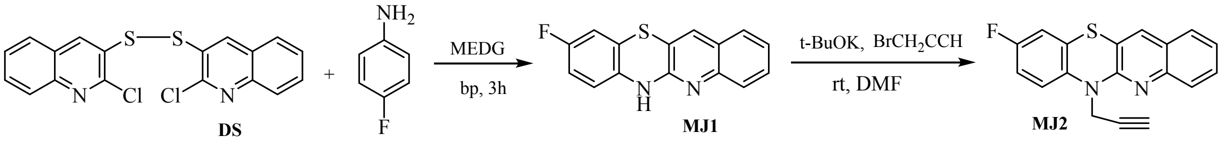

A solution of disulfide (**DS**) (0.20g, 0.5 mmol) and *p*-fluoroaniline (2 mmol) in MEDG (5 mL) was refluxed for 3 h. After cooling the solution was poured into water (20 mL) and alkalized with 5% aqueous sodium hydroxide to pH = 10. The resulting solid was filtered off, washed with water and purified by column chromatography (silica gel, $CHCl_3$) to give 6*H*-9-fluoroquinobenzothiazines **MJ1** (0.13 g, 49%), mp 158-159 °C (EtOH). ^1H NMR ($CDCl_3$) δ: 6.58 (d, 1H, H7), 6.75 (m, 2H, H8, H10), 7.26 (m, 1H, H2), 7.49 (m, 2H, H1, H3), 7.56 (m, 1H, H4), 7.57 (s, 1H, H12). EI MS m/z: 268 (M, 100), 236 (M-S, 60). Anal. Calcd. for $C_{15}H_9FN_2S$: C 67.15, H 3.38, N 10.44. Found: C 67.01, H 3.39, N 10.21 [2].

To a solution of 6*H*-9-fluoroquinobenzothiazine (**MJ1**) (0.13 g, 0.5 mmol) in dry DMF (5 mL) potassium tert-butoxide (0.080 g, 0.72 mmol) was added. The mixture was stirred at room temperature for 1 h. Then 80% solution of propargyl bromide in toluene (0.15 mL, 1.35 mmol) was added dropwise. The solution was stirred at room temperature for 24 h and poured into water (20 mL), extracted with methylene chloride (20 mL), dried with Na_2SO_4 and evaporated to the brown oil. The residue was purified by column chromatography (silica gel, $CHCl_3$) to give 9-fluoro-6-propargyl-quinobenzothiazine (**MJ2**) (0.12 g, 78%), mp 124-125 °C. ^1H NMR ($CDCl_3$) d: 2.31 (s, 1H, CH), 4.95 (s, 2H, CH_2), 6.91 (m, 2H, H-7, H-8) 7.20 (m, 1H, H-10), 7.30 (t, 1H, H-3) 7.50 (t, 1H, H-3), 7.54 (d, 1H, H-1) 7.68 (s, 1H, H-12), 7.82 (d, 1H, H-4). ^{13}C NMR ($CDCl_3$) d: 35.78 (CH), 72.24 (C), 79.75 (NCH_2), 113.72 (d, J_{C-F} = 25.5 Hz, C-10), 113.97 (d, J_{C-F} = 22.5 Hz, C-8), 116.74 (d, J_{C-F} = 8.3 Hz, C-7), 117.21 (C-11a), 122.20 (d, J_{C-F} = 8.3 Hz, C-10a), 124.60 (C-2), 126.02 (C-12a), 126.25 (C-1), 127.66 (C-4), 129.38 (C-3), 132.25 (C-12), 137.12 (d, J_{C-F} = 2.3 Hz, C-6a), 145.60 (C-4a), 151.48 (C-5a), 158.75 (d, J_{C-F} = 242.3 Hz, C-9). FAB MS: m/z: 307 (M + 1, 100), 281 (M - C_2H_2, 30), 268 (M - C_3H_3, 70). Anal. calcd. for $C_{18}H_{11}FN_2S$: C 70.57, H 3.62, N 9.14. Found: C 70.38, H 3.60, N 8.92 [3].

References:

[1] Morak-Młodawska, B.; Pluta, K.; Latocha, M.; Jeleń, M.; Kuśmierz, D.; Suwińska, K.; Shkurenko, A.; Czuba, Z.; Jurzak, M. 10 H -1,9-Diazaphenothiazine and Its 10-Derivatives: Synthesis, Characterisation and Biological Evaluation as Potential Anticancer Agents. Journal of Enzyme Inhibition and Medicinal Chemistry 2019, 34 (1), 1298–1306. https://doi.org/10.1080/14756366.2019.1639695.

[2] Pluta, K.; Jeleń, M. Synthesis of Quinobenzo-1,4-Thiazines from Diquino-1,4-Dithiin and 2,2'-Dichloro-3,3'-Diquinolinyl Disulfide. HETEROCYCLES 2009, 78 (9), 2325. https://doi.org/10.3987/COM-09-11736.

[3] Jeleń, M.; Pluta, K.; Zimecki, M.; Morak-Młodawska, B.; Artym, J.; Kocięba, M. 6-Substituted 9-Fluoroquino[3,2-b]Benzo[1,4]Thiazines Display Strong Antiproliferative and Anti-tumor Properties. European Journal of Medicinal Chemistry 2015, 89, 411–420. https://doi.org/10.1016/j.ejmech.2014.10.070.
